# Nucleation of diamond films on heterogeneous substrates: a review

**DOI:** 10.1039/d1ra00397f

**Published:** 2021-03-10

**Authors:** Soumen Mandal

**Affiliations:** School of Physics and Astronomy, Cardiff University Cardiff UK mandals2@cardiff.ac.uk soumen.mandal@gmail.com

## Abstract

Diamond thin films are known to have properties similar to bulk diamond and have applications in both industry and fundamental studies in academia. The high surface energy of diamond makes it extremely difficult to grow diamond films on foreign substrates. Hence, to grow diamond films on non-diamond substrates, a nucleation step is needed. In this review various techniques used for diamond nucleation/seeding will be discussed. At present electrostatic seeding by diamond nanoparticles is the most commonly used seeding technique for nanocrystalline growth. In this technique the substrate is dipped in a nanodiamond solution to form a mono layer of diamond seeds. These seeds when exposed to appropriate conditions grow to form diamond layers. This technique is suitable for most substrates. For heteroepitaxial growth, bias enhanced nucleation is the primary technique. In this technique the substrate is biased to form diamond nuclei in the initial stages of growth. This technique can be used for any conducting flat surface. For growth on ceramics, polishing by diamond grit or electrostatic seeding can be used. Polishing the ceramics with diamond powder leaves small diamond particles embedded in the substrate. These small particles then act as seeds for subsequent diamond growth. Apart from these techniques, chemical nucleation, interlayer driven nucleation and mixed techniques have been discussed. The advantages and disadvantages of individual techniques have also been discussed.

## Abstract 2

Growth of diamond film on heterogeneous substrates assisted by nucleation/seeding.

## Graphical abstract

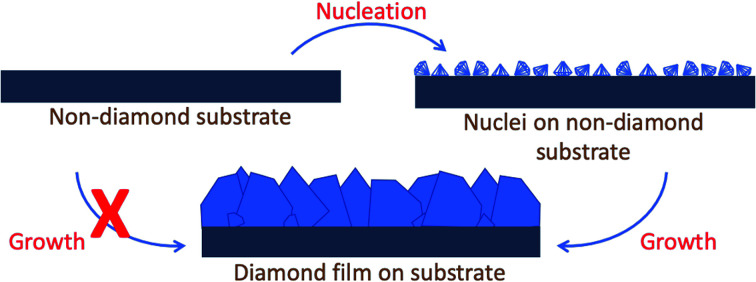

## 1 Introduction

Diamond is an allotrope of carbon with very unusual properties. It is an electrical insulator in its pure form. It also has properties like superhardness,^1^ chemical inertness^2^ and high thermal conductivity^3^ to name a few. Pure diamond is colourless and forms a clear crystal. But, small amounts of impurities can give it a blue, yellow, green, pink *etc.* colour. Its extreme properties make it ideal for the cutting and polishing tools industry. Undoped diamond has also been used within thermal and optical applications.^4^ Doped diamond on the other hand has found usage in electronics^5–8^ and device physics.^9–16^ For example, lightly boron doped diamond is a p-type semiconductor^17^ while heavily boron doped diamond is a superconductor.^18^ Electronic and superconducting devices from these materials have already been demonstrated. Furthermore, doped diamonds have also found usage in electrochemical applications.^19^ Most of these applications rely on diamonds that are grown in laboratories. Another interesting area of application for diamond is the field of quantum technologies. A brilliant news feature on diamond quantum application was written by Gibney^20^ in 2014. These quantum applications of diamond are centred around what has been described as ‘A useful hole’. The useful hole consisted of a nitrogen atom with neighbouring missing carbon atom in the diamond lattice of a natural gemstone. It was theorised that these holes, under certain conditions, can be a store for quantum information. These holes are also known as vacancy centres. The initial results published on the vacancy centres were from holes in natural diamond.^21,22^ The magic diamond was cut up and distributed amongst groups across the world. However, finding such perfect pieces of flawed diamond is extremely difficult. As a result researchers turned their attention towards lab grown diamond for such quantum applications. A detailed discussion on vacancy centres is beyond the scope of this review but authors are directed towards these articles in literature.^23–26^

Natural diamonds are formed inside the earth at depths of 150–250 kilometres. Most natural diamonds are older than one billion years. With the advancement in technology it has been possible to mimic conditions inside the earth and grow diamond in the laboratory at much shorter timescales. Systematic research in the growth of diamond under laboratory conditions started in the USA, Sweden and the Soviet Union in the 1940s. In 1955 Bundy *et al.*^27^ reported the first confirmed growth of diamond using a high pressure apparatus invented by Hall.^28^ The technique of producing diamonds by this process was known as high pressure high temperature (HPHT) technique and the diamonds were known as HPHT diamonds. The chemical properties of HPHT and natural diamonds were identical. Parallelly, research was also being conducted in the USA and the Soviet Union to grow diamonds by chemical vapour deposition(CVD). William G Eversole patented the CVD diamond growth process^29^ in 1961. Later on Angus *et al.*^30^ and Deryagin *et al.*^31,32^ were able to independently synthesise diamond using the CVD process. The CVD process is able to produce extremely pure (purer than natural) diamond which is not possible with HPHT process. Furthermore, CVD process is the most suited technique for large area thin films. Later on Spitsyn *et al.*^33^ were able to grow diamonds on non-diamond substrate using the CVD process. These initial experiments relied on spontaneous nucleation on the foreign substrates. Due to the secretive nature of research in diamond in those years the interest in CVD diamond did not catch up immediately. It was only after the detailed publication of results by Japanese groups in National Institute for Research in Inorganic Materials (NIRIM) on CVD of diamond, that interest in CVD diamond started growing. A detailed account of the early years of development can be found in the article by John Angus.^34^ From these initial years of work it was clear that fully coalesced films cannot be grown with spontaneous nucleation on foreign substrates. The subject of diamond nucleation has been studied by numerous groups over the last three to four decades and is still being pursued. As a result, a large body of literature is present on this topic and it is not possible to discuss majority of them in one single article. In this review article key nucleation/seeding techniques on non-diamond substrates will be described and possible mechanisms governing the processes will be discussed. The readers are encouraged to follow the references in this article and the references therein to develop a complete understanding of the subject.

## 2 Nucleation of diamond

Advances in growth technology have made it possible to grow diamond in laboratories. Still, spontaneous growth on large non-diamond substrates or large area growth of single crystal is not possible with the exception of iridium surfaces.^35,36^ On iridium surfaces as well an ion bombardment step is needed for nucleation to happen but still it is the best substrate available for epitaxial growth.^37^ This is due to the large surface energy difference between diamond^38^ (9.4 J m^−2^ along 〈100〉) and most common substrates like silicon^39^ (2.13 J m^−2^ along 〈100〉) and germanium^39^ (1.84 J m^−2^ along 〈100〉). This does not mean that if we were to introduce a silicon or germanium wafer in a CVD reactor there would not be any growth at all. What it means is the density of nucleation sites will be too low to generate any coalesced thin diamond film. The density of sites where spontaneous nucleation can occur is of the order of 10^4^ to 10^5^ cm^−2^.^40^ Thus to grow large coalesced thin films (<50 nm) of diamond on non-diamond substrate a nucleation density enhancement step is needed. This step is also known as seeding. Here it is important to identify a few terminologies that will be used constantly throughout the article. The first one is nucleation. From classical theory, nucleation is a thermodynamically driven process where clusters of atoms or nuclei evolve to form stable phases. The detailed theory of diamond nucleation can be found in the article by Gebbie *et al.*^41^ The second term is seeding. Seeding refers to the act of attaching/impregnating diamond particles on the surface of the substrate. This can be done in various ways and that will be discussed in the article. Thin film growth from these nucleated/seeded substrates have been reviewed by various authors which can be helpful in understanding film growth.^42–44^ The last four decades have seen extensive research in enhancing the nucleation/seeding density on non-diamond substrates. In the following subsection individual techniques of seeding will be discussed.

### 2.1 Electrostatic seeding

**Fig. 1:**
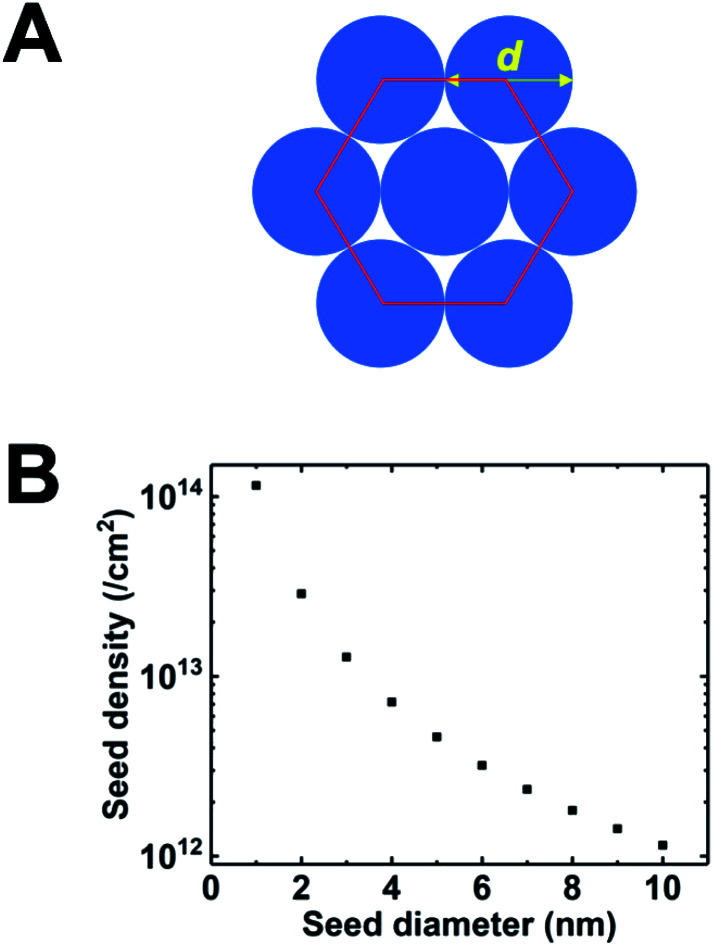
(A) Arrangement of spherical seeds in hexagonal close packed structure. (B) Maximum possible density of seeds with respect to seed diameter. The density has been calculated assuming a close packing as seen in panel A.

One of the most successful seeding techniques for growth of nanocrystalline diamond film is the electrostatic seeding technique^45^ using diamond nanoparticles. In this process the substrate is coated with diamond nanoparticles before growth. Once the coated substrate is exposed to CVD conditions the individual crystals start growing. At the beginning, the growth follows Volmer–Weber growth mechanism^46,47^ and once the islands are big enough to coalesce they follow the van der Drift columnar growth mechanism.^48^ Generally the substrates are coated with the seed by dipping in a seed solution^45^ but electrospraying^49^ and spin-coating^50,51^ have also been used. In the electrospraying method, a large potential is used to ionize liquid drop containing nanodiamond (ND) which is then accelerated towards a grounded substrate. The number of seeds deposited on the substrate during the seeding process is known as seed density. The maximum possible seed density assuming monolayer seeding can be easily calculated. If we assume that the seeds are perfectly spherical and of uniform size in the seed media, then the most densely packed seeds will have a hexagonal close packing as shown in Fig. 1A. If we join the centres of each spheres, we will get a hexagon. Each side of the hexagon is of length *d* nm. The area covered by the hexagon will accommodate only three seeds of diameter *d* nm. The area of the hexagon is 
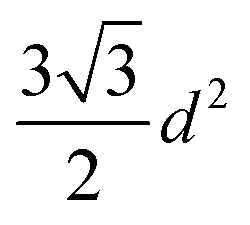
. So, the seed density would be 
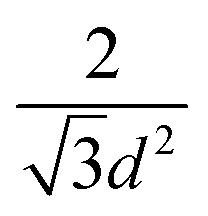
. The theoretical seed density *vs.* seed diameter is plotted in Fig. 1B. The seed diameter has been varied between 1–10 nm. The numbers that we get in the plot is a theoretical estimate. But, to experimentally detect individual particles, atomic force microscopy (AFM) is used. While AFM is extremely sensitive to height profile of a surface, its lateral resolution is limited. According to Bustamante *et al.*^52^ there is a minimum distance between two objects that can be resolved by AFM. If we assume 5 nm diameter seeds and a tip radius of 2 nm for the AFM tip then the minimum distance between two seeds has to be 6.3 nm.^52^ That makes the effective diameter of the seeds to be 11.3 nm. Now if we calculate the theoretical density based on the effective diameter it comes to 9 × 10^11^ cm^−2^. Implied within this is the assumption that all seeds are of uniform size and shape which is not the case in most seeding media. So a seeding density of 5–8 × 10^11^ cm^−2^ is more reasonable to be detected by conventional techniques like AFM. Higher seeding densities may be detected by using techniques like spectroscopic ellipsometry.^53^

A brief comment about the diamond solution is also important here. Diamond is not soluble in water or any other solvent. So diamond nanoparticles form a colloidal suspension when added to water or any other solvent. The stability of any colloidal suspension is related to the *ζ*-potential of the suspended particles. A *ζ*-potential value outside the −30 mV to +30 mV range is regarded optimal for stable colloid.^54^ However this does not mean that large quantities of nanodiamond can be added to a solvent. As the concentration of the particles go up so does the chance of collision of the particles. With higher chance of collision it is possible that the attractive van der Waals forces between particles overcomes the repulsive potential due to surface charges. Furthermore, the stability of the colloid is also effected by particle size.^55^ In depth discussion about colloidal stability is beyond the scope of this review. So, for efficient seeding it is important to start with a stable monodispersed ND suspension. Methods for preparing such suspensions can be found in these articles.^56–58^ Also important is the surface charge or *ζ*-potential of the substrate once it is immersed in the diamond solution. The seeding of substrates by diamond nanoparticles is driven by electrostatic forces between substrate and diamond particles. Hence the knowledge of the *ζ*-potential of the substrate is important for optimal seed density. A discussion on the substrate *ζ*-potential and its effect on seeding will be done later in this subsection.

**Fig. 2:**
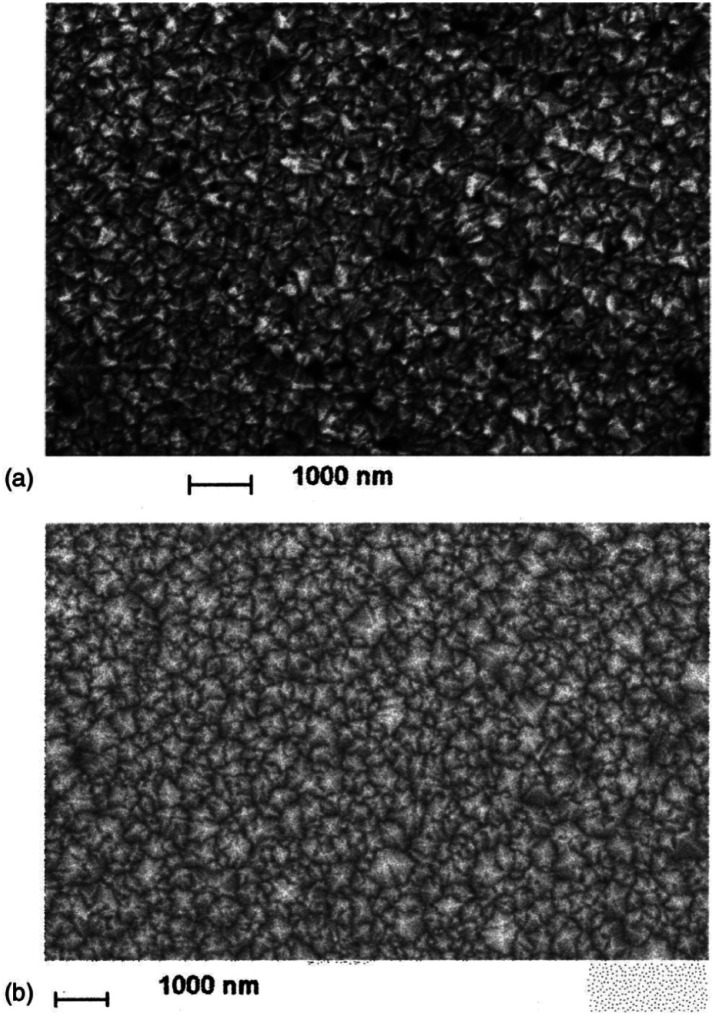
SEM images of diamond films grown on DND seeded silicon wafers. The two wafers were grown a few months apart using different seed solutions. (Reprinted with permission from Philip *et al.*, *J. Appl. Phys.*, 2003, **93**, 2164, AIP Publishing.^66^)

**Fig. 3:**
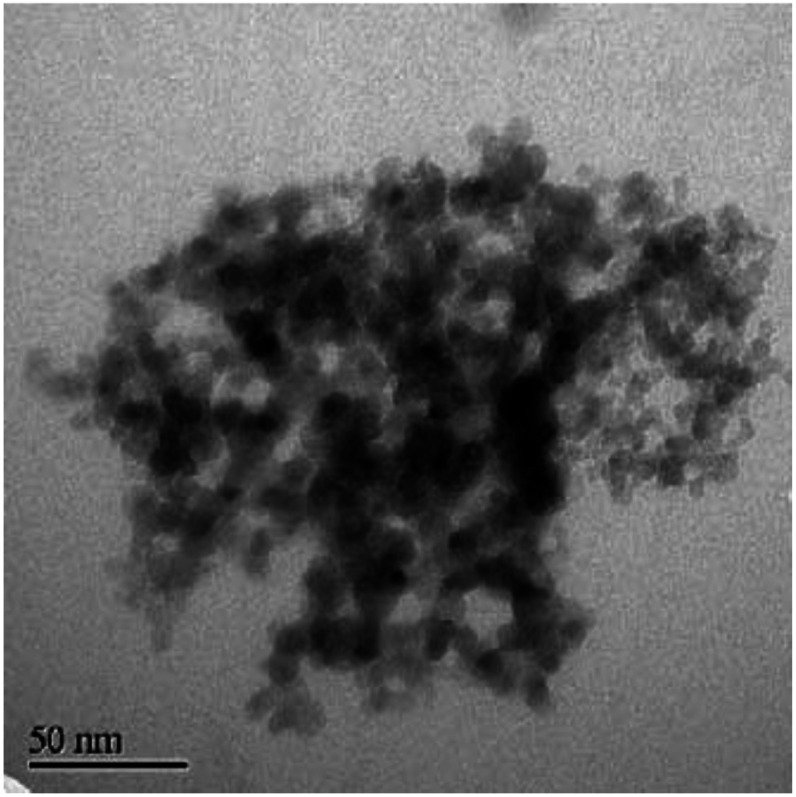
TEM image of 5 nm diamond powder. Aggregation of the powder can be clearly seen in the image. (Reprinted with permission from Daenen *et al.*, *Phys. Status Solidi A*, 2006, **203**, 3005, John Wiley and Sons.^69^)

The earliest attempt to use ND for diamond growth was done by Geis.^59^ ND seeds were attached to a substrate and plasma technique was used to grow the seeds into 10 micron size diamond. At the time when this work was done only large size diamond particles were commercially available. Smaller particles which are currently used for seeding purposes are produced by detonation of unused or decommissioned explosives and hence are called detonation nanodiamond (DND). The history and discovery of DND is quite interesting and readers can look into these book chapters by Danilenko^60^ and Petrov *et al.*^61^ for more details. On a lighter note, The SKN Company [RUSSIA] has won Ig Nobel for converting old Russian ammunition into new diamonds.^62^ DNDs started becoming available more freely in the early 2000s. During the process of ND formation by detonation,^63^ considerable amount sp^2^ carbon is also formed.^64,65^ The non-diamond carbon binds the diamond particles in large agglomerates ranging in sizes greater than 100 nm. Such agglomerates cannot give rise to high seeing density (see Fig. 1). Philip *et al.*^66^ used DNDs to seed silicon substrates and were able to get coalesced diamond films. They used a combinatorial technique developed by Rotter and coworkers (discussed later)^67,68^ to seed their substrates. Fig. 2a and b shows the ND films grown by Philip *et al.*^66^ The films were grown few months apart. The poor quality of the film in Fig. 2a has been attributed to poor seed solution. The authors have claimed a seeding density in excess of 10^12^ cm^−2^, which is overestimated (see Fig. 1) considering the seed solution consisted of large grains of agglomerated DNDs. Later on Daenen *et al.*^69^ compared mechanical abrasion and ultrasonic seeding technique. With optimisation they were able to achieve seeding densities upto 10^11^ cm^−2^. Additionally they also observed that ND powder formed large agglomerates when added to methanol which was used as seed solution. The TEM image of an agglomerate is shown in Fig. 3. Even though Daenen *et al.*^69^ were able to achieve high seeding density the seed solution was not ideal for seeding. With the presence of 200 nm agglomerates it would be impossible to get very thin diamond films which is not the case for seeding with 5 nm seed solution.^70^

**Fig. 4:**
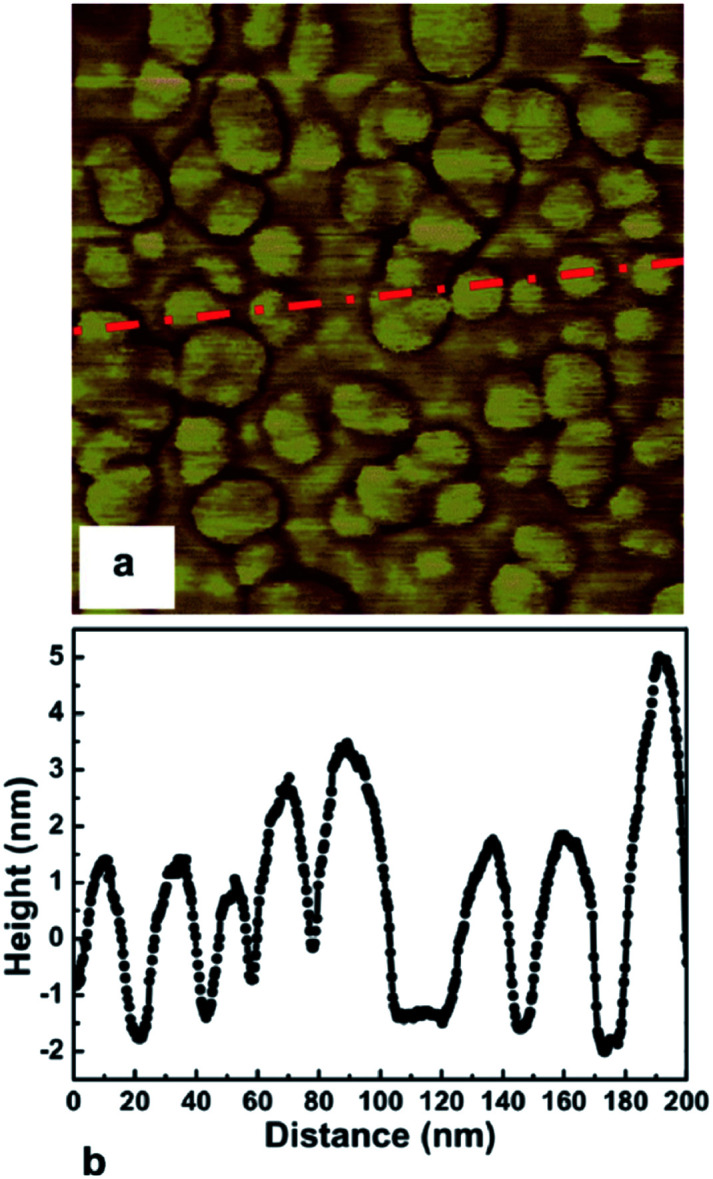
AFM image of seeded silicon surface (200 nm × 200 nm) is shown in Panel a. Panel b shows a line trace from the AFM data indicated by red line in panel a. (Reprinted with permission from Williams *et al.*, *Chem. Phys. Lett.*, 2007, **445**, 255, Elsevier.^45^)

The first mono dispersed solution from DNDs containing 4–5 nm diamond particle was prepared by Ozawa *et al.*^71^ by a wet milling process using zirconia beads. It was also found that the *ζ*-potential of the colloids were in the range of +45 to +50 mV resulting in stable colloids. The pH value for the measurement has not been explicitly mentioned in the work but it can assumed to be between 6 and 7 since the milling has been done in deionised water. This is opposite to the *ζ*-potential seen for DND solutions which is negative (pH ∼ 9.5–10) in nature^72^ due to the oxygenated terminations of the DNDs.^73,74^ A solution of mono dispersed diamond particles created by the milling method^71^ was used by Williams *et al.*^45^ to enhance diamond nucleation on silicon substrates. They reported a seed density in excess of 10^11^ cm^−2^. The seed density was calculated from the AFM data. The AFM data for the seeded silicon surface in shown in Fig. 4a and b, which shows a 200 nm × 200 nm area. Fig. 4a is the AFM image and Fig. 4b is a line cut on the image as indicated by the red line. As discussed earlier, AFM has excellent height sensitivity but its lateral sensitivity is not that good. We can clearly see seeds on the surface of the silicon but the individual seeds are not resolved accurately. Assuming the seeds are near spherical in nature we can estimate the size from the height profile. The line trace showing the height profile is shown in Fig. 4b. It shows heights ranging from 5–7 nm which is also the spread of the diamond nanoparticle size in the seed solution.^45^ In the work the authors made a comment about maximum seed density using square closed pack configuration, which has been discussed in this review using hexagonal close packing configuration (see Fig. 1). Unfortunately, in the literature there are many reports of achieving seed densities in excess of 10^12^ cm^−2^ with +5 nm ND seeds. Such high density numbers are mere guess work and should be taken cautiously (see discussion with Fig. 1 earlier). In some cases such numbers have been inferred from diamond grain density after certain amount of growth. It should be kept in mind that secondary nucleation is also possible under certain conditions giving rise to a sense of enhanced nucleation density. In general when seed density is talked about, it refers to the number of seeds on the substrate prior to growth. However, it is worth mentioning that if the seed sizes can be reduced, high seed density can be achieved. Stehlik *et al.*^75^ showed nucleation densities in excess of 10^13^ cm^−2^ while using 2 nm DND seeds. The theoretical seed density, while using 2 nm seeds, can be as high as 2.88 × 10^13^ cm^−2^ (see Fig. 1) where all the seeds are touching the substrate.

The following year Arnault *et al.*^76^ studied the stability of the ND seeds on silicon surfaces under intense hydrogen plasma. They found that the seeds were able to survive in temperatures as high as ∼1200 K. In contrast, when the ND seeds on silicon surface were annealed in ultra high vacuum conditions they could only survive at temperatures below 1100 K.^77^ The survivability of ND seeds in hydrogen plasma at temperatures greater than 1100 K has been attributed to a passivation layer formation by hydrogen plasma.^77^ The higher survivability of NDs in hydrogen plasma should be taken with caution, as pyrometers, used for reading of temperature in the plasma study,^76^ can give large errors in reading if not properly used. Even then these studies confirmed that ND seeds can be used to grow diamond films over a wide temperature range. Initial works on electrostatic seeding was done by using water or methyl alcohol as dispersant. Scorsone *et al.*^50^ added small amounts of polyvinyl alcohol to ND/water colloid. The polymer would form a thin layer on the substrate with NDs embedded in the matrix. By varying the concentration of polymer in the solution the authors were able to control the seed density as well. This technique may work on any kind of surface as the particle–polymer matrix is spin coated onto the substrate. Even then it has its own disadvantage, the simple electrostatic seeding, discussed earlier, forms a mono layer of seeds on the substrate which is unlikely to be achieved by particle–polymer matrix^50^ or the electrospraying method.^49^ A similar approach to Scorsone *et al.*^50^ as used by Daenen *et al.*,^78^ but in this case the ND seeds were not mixed in the coating material. Daenen *et al.*^78^ and later Lu *et al.*^79^ deposited a sol–gel TiO_2_ layer on ND seeded and unseeded substrates. Some of the substrates were also coated with ND seeds on its own.^78^ CVD growth conditions were applied to all three types of treated surfaces. It was found that irrespective of the seeds being present on top or bottom of the TiO_2_ layer, diamond thin films were obtained. The sol–gel TiO_2_ coated unseeded substrate showed very low nucleation density. The point to be noted here is even if the diamond cores are buried under thin layer of material it can still generate nucleation sites and that it is essential to have seeds for growth of diamond on non-diamond substrates.

**Fig. 5:**
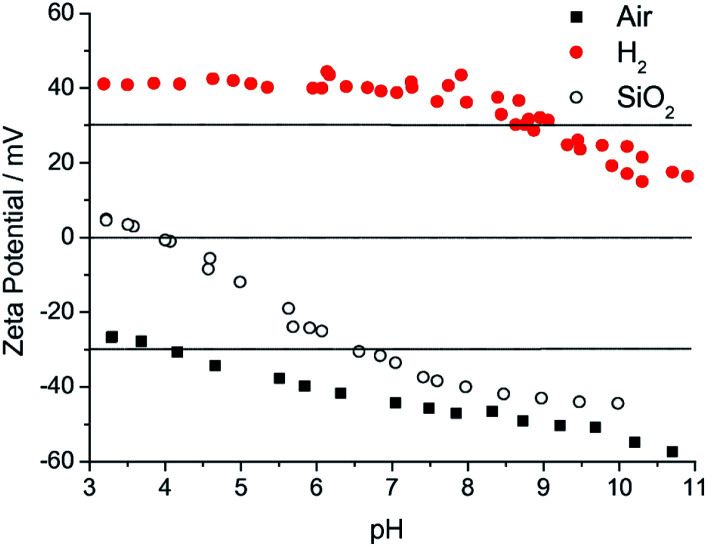
*ζ*-Potential of hydrogenated (H_2_) and oxygenated (air) diamond colloid measured over a wide range of pH. Also shown is the *ζ*-potential of a silicon dioxide surface. It is clear that for electrostatic seeding on silicon surfaces containing naive oxide, hydrogenated diamond seeds will lead to higher seed density. (Reprinted with permission from Hees *et al.*, *Chem. Phys. Lett.*, 2011, **509**, 12, Elsevier.^57^)

A brief discussion about what is driving the high seeding density with milled particle solution is important here. In the beginning of this subsection, *ζ*-potential was briefly mentioned regarding stability of the colloid. This *ζ*-potential is also important for seeding. Any surface when submerged in a liquid will form an electric double layer.^80,81^ The first layer consists of ions that are adsorbed on to the surface due to chemical interactions. The second layer consists of ions that are attracted to the first layer and electrically screens the first layer. This second layer is also known as diffuse layer. The diffuse layer can partially move with the liquid. There is an interface which separates the mobile liquid with the liquid attached to the solid surface. This interface is known as slipping plane and the electric potential at the slipping plane of the double layer is known as *ζ*-potential. The *ζ*-potential of the milled nanodiamond solution is positive^71^ and the potential for the silicon dioxide is negative^57^ in pH range of 4–10 (Fig. 5). As soon as the silicon (a native oxide layer is always present on silicon surface) substrate is dipped in the ND colloid, the positively charged particles get attracted towards the negatively charged silicon surface. This electrostatic interaction drives the self assembly of ND monolayer on the substrate.^57^ The colloid prepared by the method described by Ozawa *et al.*^71^ has positive *ζ*-potential and contains some amount of zirconia contaminants.^45,71^ That means only substrates that have a negative *ζ*-potential in water can be seeded with this solution. Also the ND colloid prepared from commercially available ND without any treatment results in large particle size in the colloid.^56^ To solve this problem Williams *et al.*^56^ devised a new technique to prepare monodispersed ND colloid with particle sizes between 4–7 nm. The *ζ*-potential can also be tailored to be either negative or positive with values outside the −30 mV to 30 mV range. Fig. 5 shows the *ζ*-potential of both hydrogenated and oxygenated diamond colloid solution. More details about the methods for making these colloids can be found in these articles.^56,57,82^ While Chakrapani *et al.*^82^ showed the positive *ζ*-potential in 0.5 and 1 μm diamond grit exposed to hydrogen plasma, Williams *et al.*^56^ showed the same in 4–5 nm nanodiamond annealed in hydrogen atmosphere. The readers are guided towards these articles^82–87^ for mechanism of hydrogen termination of NDs and the possible explanation for the positive *ζ*-potential.

The availability of diamond colloids with either polarity meant that any kind substrates could be seeded with this technique. Some researchers have also tried to switch the *ζ*-potential of the substrate by coating it with a polymer layer so as to realise a *ζ*-potential opposite to the ND colloid.^89,90^ The polymer layer is etched away during CVD growth leaving the ND seeds behind. Over the years many researchers have studied the seeding mechanism, seed solution effect, effect of *ζ*-potential *etc.* on diamond seeding. We will discuss a few of those studies in the following few paragraphs. In a study by Yoshikawa *et al.*,^91^ it was found that the diamond seeds have tendency to form agglomerates on the substrate. They reported that small amount of potassium chloride can help to reduce such agglomeration. But in reality such aggregation can happen due to variety of reasons from unclean substrates, low *ζ*-potential of substrate surface *etc.* So, for good seeding the following things are important, (i) monodispersed ND colloid, (ii) knowledge of *ζ*-potential of colloid, (iii) knowledge of *ζ*-potential of substrate and (iv) clean substrate surface.

**Fig. 6:**
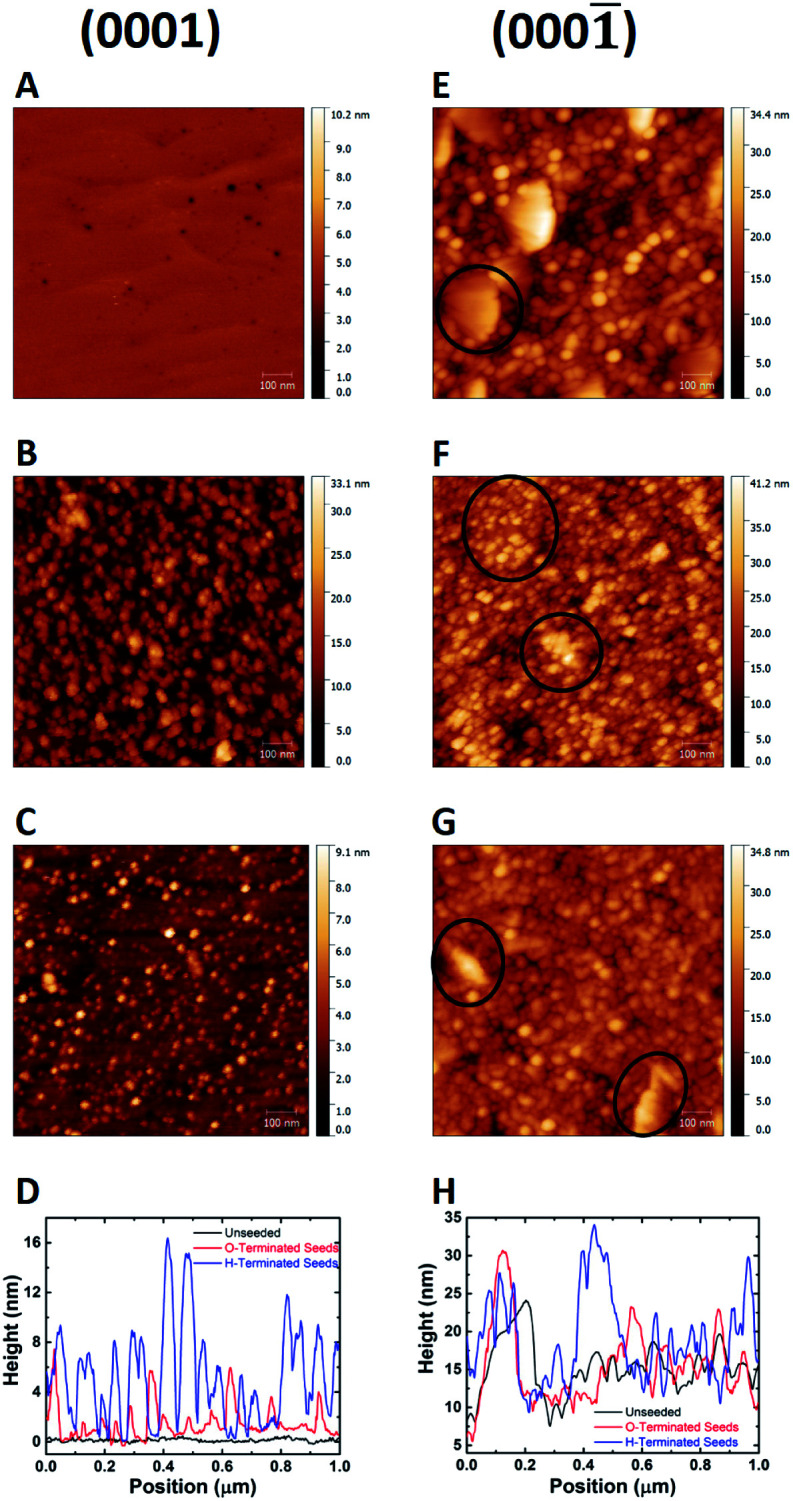
AFM images of the Ga(0001) and N(0001̄) faced GaN seeded with hydrogenated (panels B and F) and oxygenated (panel C and G) diamond seeds. The unseeded surfaces are shown in panels A and E. Panels D and H show the line traces from the AFM images. (Reprinted with permission from Mandal *et al.*, *ACS Omega*, 2017, **2**, 7275, American Chemical Society.^88^)

The technique of seeding with ND colloid have been applied to variety of surfaces. For example, by measuring the *ζ*-potential of the gallium nitride surface^88^ researchers have been able to grow diamond on both nitrogen and gallium polar surfaces. It was found that the *ζ*-potential has a strong dependence on the adsorbed oxygen on the substrate. The *ζ*-potential of GaN surface was found to be negative over a wide range of pH for both polarities. Fig. 6b, c, f and g shows the AFM images of the seeded surfaces. To begin with, N-faced (0001̄) (Fig. 6e) GaN surface has a rough topography when compared with Ga-faced (0001) (Fig. 6a) GaN. However, one can easily spot the high density of diamond seeds when the surfaces were treated with hydrogenated diamond colloid (Fig. 6b and f). The images in Fig. 6c and g show the surfaces seeded with oxygen terminated diamond seeds. The marked areas in Fig. 6e–g are to show the difference on surface of large grains in the film. The line traces in panels d and h reveal a monolayer of seeds on the surface. The main aim of these growth were to use diamond for thermal management in high-power GaN devices. For proper thermal management a thick (≥50 μm) layer of diamond is needed. Even though with high seed density a coalesced thin diamond layer could be formed, a thick diamond layer could not be realised. More detailed explanation for this can be found here.^92^ The point being, just good seeding is not enough, the main purpose of the diamond film is also a consideration for seeding. In a recent work it was shown that low seeding density is beneficial for growth of thick diamond layer on aluminium nitride^92^ for thermal management applications.

**Fig. 7:**
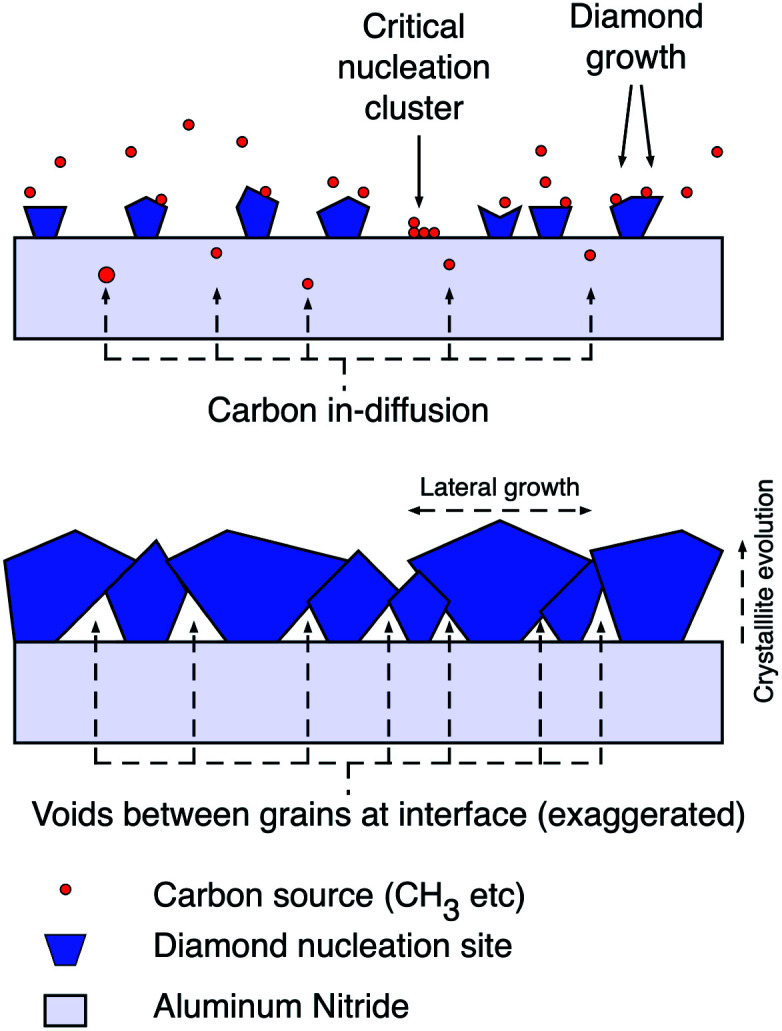
Diamond growth schematic with low seed density indicating a mixture of diamond growth on seeds and carbon in-diffusion in to the substrate during initial stages of growth. (Reprinted with permission from Mandal *et al.*, *ACS Appl. Mater. Interfaces*, 2019, **11**, 40826, American Chemical Society.^92^)

The aluminium nitride surface showed similar trends in *ζ*-potential as the GaN surface. But, a plasma pre-treatment with a mix of nitrogen and hydrogen gas was needed for creating thick adherent diamond layer. The pre-treatment increased the oxygen content of the surface and showed lower *ζ*-potential.^92^ If we look at the *ζ*-potential of aluminium oxide particles it is positive^93,94^ but sapphire plates show negative *ζ*-potential^95^ in the pH range reported by Mandal *et al.*^92^ Hees *et al.*^96^ claimed the AlN surface to have positive *ζ*-potential based on their measurements on AlN nanoparticles rather than AlN plates. It is possible that the AlN particle surface is heavily oxidised and thus shows positive *ζ*-potential. So, it is important to measure the *ζ*-potential of the proper shape of the material as it is a strongly surface dependent quantity and surface terminations are heavily effected by shape and size of material. As expected in the case of Mandal *et al.*,^92^ the surface when seeded with hydrogenated seeds led to high seed density and the opposite for oxygenated seeds. In this case the high seed density was not beneficial for the work. The thick diamond layer peeled off immediately after growth. The most probable reason is the stress induced by the thermal mismatch between substrate and diamond film as the growth happens at an elevated temperature of 800 °C.^97^ When the seed density is lower, voids were created in the initial layers of diamond. These void layers may have resulted in reliving some of the stress in the film. This did not effect the thermal boundary resistance between diamond and AlN film which was the main aim of the work.^92^ In Fig. 7 a schematic explaining growth with low seeding density is shown. It is believed that in the areas where diamond seeds are absent some in-diffusion of carbon takes place with carbon accumulation on the surface. These surface carbons are mostly non-diamond in nature. As the seeds grow bigger and coalesce, the non-seeded areas are blocked from the plasma and the voids are enclosed at the interface. Raman measurements on the initial growth phase reveals high non-diamond carbon in the material validating the postulate.^92^

It is clear from the above result that low seeding density is beneficial for certain cases and that it is also possible to get fully coalesced films with low seed density. If we consider a hypothetical situation where we have a seed density of 10^10^ cm^−2^ of perfectly spherical seeds of 5 nm in diameter, the distance between the seed centre in a hexagonal close packing configuration will be ∼110 nm (see Fig. 1). Furthermore, if we assume that lateral and longitudinal growth rates are same for the seeds, a coalesced film will be formed within 100 nm without any spontaneous nucleation and much before with spontaneous nucleation. Now if we look at this film with SEM, it may give an impression of seeding densities in excess of 10^11^ cm^−2^. Hence, it is important that if any conclusive information is to be given for seed density it has to be done before any growth. For nucleation sites created by chemical route, like with adamantane, techniques like ellipsometry is more reliable.

In the work on AlN^92^ a plasma pre-treatment has been used to create adherent layer. Plasma treatments have also been used in the past on other surfaces to facilitate electrostatic seeding.^98–103^ This should not be confused with the combinatorial seeding technique (discussed later)^67,68,104^ in which a thin carbon layer is formed on the substrate before electrostatic seeding. The measured *ζ*-potential of any surface gives the average over certain area or certain volume of liquid at a given pH. It is possible that even if the total potential is negative there are large areas where the *ζ*-potential is positive. When this substrate is dipped in a diamond solution this may not result in good seed density. Bland *et al.*^102^ have showed that exposure to oxygen plasma makes the *ζ*-potential of silicon nitride surface more negative thus helping in the seed density. They found that even after cleaning the silicon nitride surface with RCA-1 clean, pinholes were seen in the diamond films grown on the cleaned substrates contrasting the pinhole free diamond film on oxygen plasma exposed silicon nitride surface.^102^ Similar results have been seen for other surfaces like silicon dioxide, silicon, silicon carbide to name a few. The nanoseeding technique is now being routinely used to grow diamond on ceramics,^105,106^ cutting tools^107–110^*etc.* Researchers have also used ND solutions for selective seeding using ink-jet style writing technology,^111–114^ contact printing,^115^ photolithography^116–118^ or masking^116,119,120^ to define diamond structures without post diamond growth lithography and etching. The size dependence of the seed solution has also been investigate by some groups.^89,121^ The technique of seeding by ND solution is extremely versatile and should be adapted based on the application of the final diamond films.

### 2.2 Bias enhanced nucleation

**Fig. 8:**
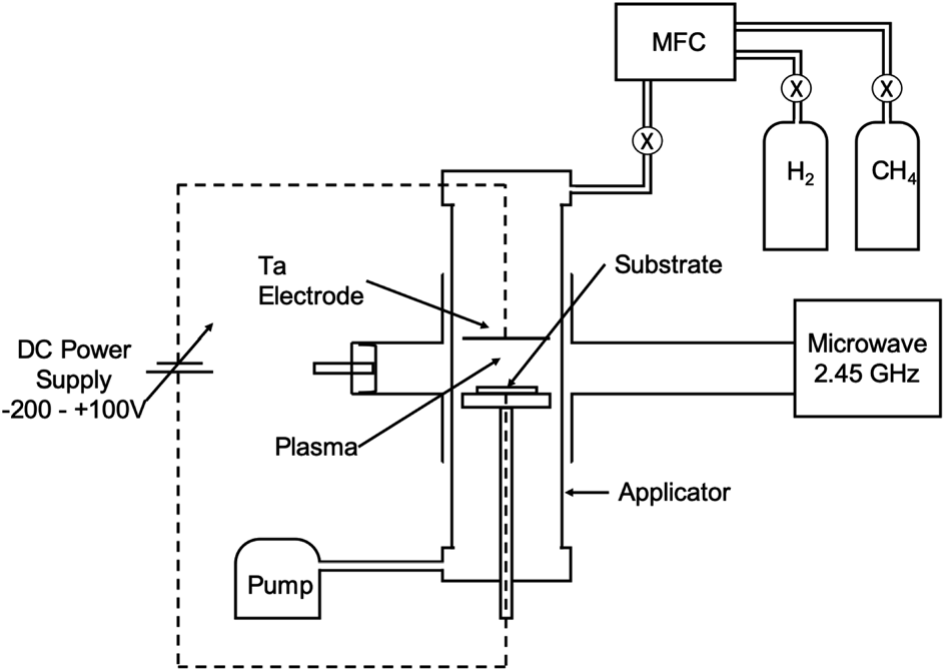
Schematic of the CVD reactor, showing the biasing electrodes, used by Yugo *et al.*^122^. (Reprinted with permission from Yugo *et al.*, *Appl. Phys. Lett.*, 1991, **58**, 1036, AIP Publishing.^122^)

**Fig. 9:**
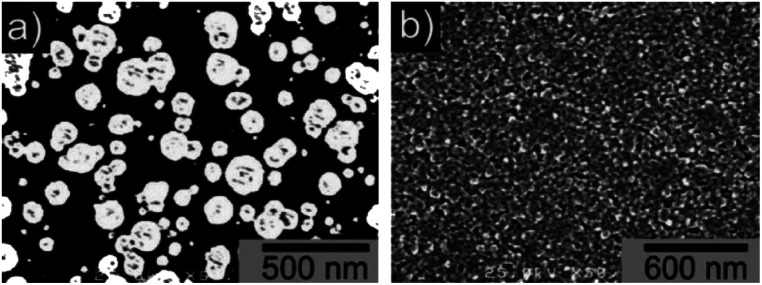
High resolution SEM images of substrate surface after two different BEN steps. Panel A is for BEN1 and Panel B is for BEN 2. A clear enhancement in nucleation density can be seen for the BEN 2 in panel b. Details about BEN 1 and BEN 2 are given in the text. (Reprinted with permission from Arnault *et al.*, *Chem. Vap. Deposition*, 2008, **14**, 187, John Wiley and Sons.^125^)

**Fig. 10:**
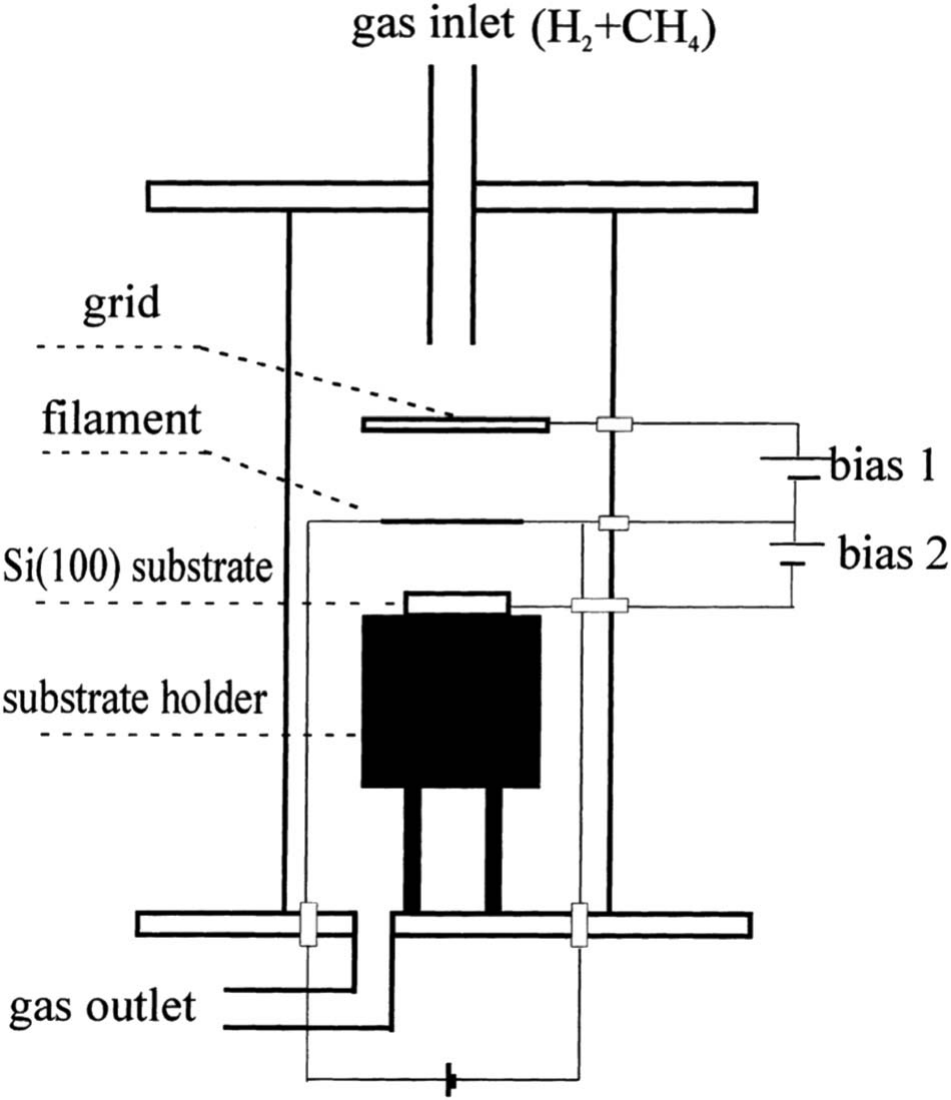
Schematic of the CVD reactor, showing the double biasing electrodes, used by Zhou *et al.*^127^ (Reprinted with permission from Zhou *et al.*, *Diamond Relat. Mater.*, 2000, **9**, 134, Elsevier.^127^)

Nucleation of diamond using a bias voltage during the CVD process, also known as Bias Enhanced Nucleation (BEN), was first demonstrated by Yugo and co-workers.^122,123^ It is worth mentioning that while discussing BEN we will talk in terms of nucleation/nucleation density rather than seed/seed density. Nucleation density is determined by counting the crystallites which have passed the critical size, thus a real nuclei is never counted. In this process a pre-deposition step was introduced. The pre-deposition step consisted of a high methane content (upto 40% CH_4_/H_2_) plasma along with an applied bias to the substrate. To achieve this, a spiral of tantalum wire was placed 4 cm away from the substrate. The substrate was biased with respect to the grounded tantalum wire. Once the pre-deposition step was over the biasing was removed and normal growth process conditions were set. The schematic of the CVD setup used by Yugo *et al.*^122^ is shown in Fig. 8 showing the grounded Ta wire and the biased substrate. This nucleation (not seeding) process achieved a nucleation density of 10^10^ cm^−2^. Higher nucleation densities of 10^11^ cm^−2^ have been reported by Stoner *et al.*^124^ and later by Arnault *et al.*^125^ To achieve this, Arnault *et al.*^125^ changed the parameters in the pre-deposition phase of the growth. Fig. 9 shows the high resolution SEM images of the substrate surface after two different BEN treatments. BEN1 (Fig. 9a) was done with 550 W microwave power, 18 mbar gas pressure, 1123 K temperature, −250 V bias voltage, 3% methane and 18 minutes step time. The condition for BEN2 (Fig. 9b) were, 450 W, 30 mbar, 1023 K, −307 V, 10% and 5.5 minutes. A clear increase in coverage can be seen for the BEN treatment in panel b. Though the authors have reported some numbers to the nucleation density, it is not possible to get nucleation density from SEM images. It can only be inferred as the crystallite sizes are expected to be very small, in the range of few nanometers. The first treatment is similar to the ones used for epitaxial growth^125^ which leads to nucleation density of around 10^9^ cm^−2^. A slight lowering of plasma power, gas pressure along with increased negative substrate bias leads to the high nucleation density condition. BEN was initially demonstrated in a microwave CVD system, but the technique can also used in hot filament CVD systems. In hot filament system, the insufficient generation of charge carriers leads to low current under low bias condition.^126^ Zhou *et al.*^127^ developed the double bias-assisted technique to induce nucleation at lower bias voltages/currents. In this technique an extra electrode was introduced between filament and substrate. A schematic of the CVD system with double bias is shown in Fig. 10.

It is true that this method greatly improved the seeding density for diamond growth thus paving the way for high quality nanocrystalline diamond films, the major drawback of this technique is its applicability to electrically conducting substrates only. However, having a conducting surface is not the sufficient condition. Wolter *et al.*^128^ found that while BEN increased nucleation on silicon surfaces, it was not effective for polycrystalline copper surfaces. The authors attributed this to the ease with which carbide can be formed on the silicon surface as compared to copper. Stoner *et al.*^124^ studied the substrate surface by interrupting the pre-deposition at different times and found the carbide formation alone is not sufficient. It was found that majority of carbon on the silicon surface was in the form of silicon carbide with only 20% of bonds attributed to C–C bonding for bias times between 5 min and 1 hour and in this duration no diamond seeds were detected. The excess carbon on the surface is due to the evaporation of silicon from the surface silicon carbide. When the bias time was increased beyond 1 hour, and only then an increase in C–C bond was observed and signature of diamond was seen for bias times of 1.5 hours and above. It should be noted here that these biasing experiments were carried out at 2% CH_4_/H_2_ conditions, which are much lower than what was reported by Yugo^122^ and Arnault.^125^ Overlayer calculations done by Stoner *et al.*^124^ for the biasing pre-treatment revealed a silicon carbide layer saturation at 4.2 nm. The interesting thing to note here is that the silicon carbide layer is 9 nm thick at the end of 1 hour pre-treatment and then it starts etching away finally saturating at 4.2 nm. The authors calculated that the highest seed density was achieved for a biasing time of 2 hour *i.e.* a stable silicon carbide layer is present. If the biasing step was carried out beyond the 2 hour limit, then the resulting film was of much poor quality. This should point to the fact that silicon carbide plays a major role in nucleation. By extension, pure silicon carbide substrates or silicon substrates covered with silicon carbide should give enhanced nucleation. However, such nucleation enhancement was not seen on silicon carbide by Hartnett *et al.*^129^ Reiterating that just silicon carbide formation is not enough for nucleation enhancement. Stoner *et al.*^124^ argued that the enhancement in nucleation density is the result of combination of factors during the pre-deposition step of which formation of silicon carbide is only a part. They further concluded that the conditions for formation of diamond nucleus is very different from the growth regime. Csencsits *et al.*^130^ saw similar results to Stoner *et al.*^124^ in their pre-deposition stages of growth. They claimed through high resolution transmission electron microscopy that no diamond seeds were formed in the bias step. It was only after the bias step that diamond seeds start appearing. This may be partially true since the small diamond nucleus will be surrounded by non-diamond carbon and the diamond core may be extremely difficult to detect. These non-diamond layers are etched away once the growth stage starts and the diamond nucleus starts to grow giving an impression that the initial stages have no diamond at all. The presence of small amounts of diamond after pre-treatment stage has been shown by Stoner *et al.*^124^ in their work using Raman spectroscopy.

Now the obvious question to ask is the effect of bias voltage on the seeding process. Many researchers^122,123,131–136^ have reported on the effects of biasing, both positive and negative. The bias voltage applied during the pre-deposition follows different routes for nucleation enhancement depending on whether the bias is negative or positive. The positive bias leads to a electron shower effect on the substrate while negative bias leads to an ion shower.^137,138^ Yugo *et al.* varied the bias voltage between −200 V and 100 V.^122,123^ They found that no nucleation was observed for bias between 100 V and −50 V. The nucleation only started at bias voltage of −70 V with the highest nucleation at −100 V. This bias dependence was done at 40% CH_4_/H_2_ concentration. By fixing the bias voltage at −100 V the methane concentration was varied. It was found that the highest density was obtained when the concentration was 10%.^122^ Similar results were obtained by Jiang *et al.*^131^ as well. In addition to studying the effect of bias voltage on seeding density, they also looked into its effect on the morphology of resulting growth. Spherical particles with diamond facets were found when the bias voltage was between 0 and −60 V. Drastic increment in nucleation density was seen at −70 V like before^122^ but the transition to diamond crystals only happened when the bias was beyond −80 V. All these results point to a very strong dependence of the bias voltage, implying that only when the ion energy is above a threshold it contributes to diamond nucleation. Gerber *et al.*^132^ studied the ion energy distribution in the pre-deposition plasma. It is to be noted that the substrate is placed at a distance from the plasma ball. This means that the inelastic collisions between the accelerated ions cannot be overlooked. So, the ion energies may not be identical to applied voltage. Gerber *et al.*^132^ found that maximum nucleation happened at ion energies of ∼90 eV. They concluded that the nucleation density is a strong function of the sp^3^ bonding fraction in deposited carbon material. This seems an obvious statement but the formation of sp^3^ or non-diamond bonds are dependent on the incident ion energies. Building on the theoretical work by Lifshitz^139^ and Robertson^140^ they concluded that the nucleation is driven by subplantation of C-atoms to form sp^3^ carbon clusters.

**Fig. 11:**
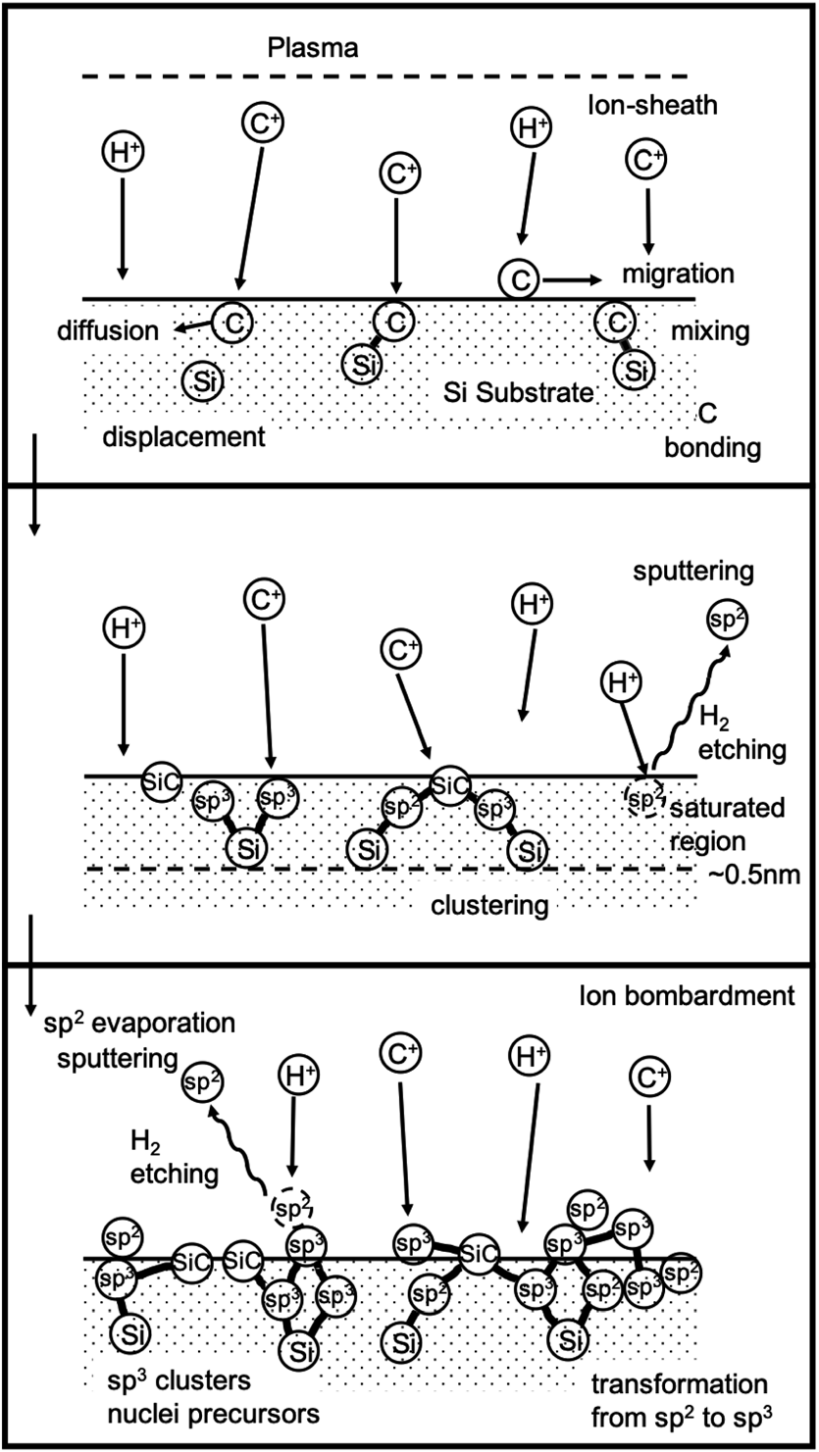
Schematic of the model given by Yugo *et al.*^141^. (Reprinted with permission from Yugo *et al.*, *Diamond Relat. Mater.*, 1993, **2**, 328, Elsevier.^141^)

At this point it is important to understand the mechanism behind BEN and this has been described by few researchers.^141–146^ Yugo *et al.*^141^ gave a model for BEN based on ion impinging effect. They claimed that the energy of ions impinging upon the substrate surface is proportional to the ratio of sheath width to mean free path (top panel Fig. 11). Based on this ratio Yugo *et al.*^141^ claimed the ion energies to be nearly equivalent to bias voltage contrary to the claims by Gerber *et al.*^132^ later on. Clusters containing both diamond and non-diamond fragments are formed when carbon ions land on substrate surface (middle panel Fig. 11). This clustering process can be slowed down by etching and diffusion of the carbon atoms. For diamond growth to happen the clusters should have more sp^3^ components then non-diamond components. If the ion energies reaching the surface can be tailored, by application of bias, to preferentially etch weakly bonded non-diamond^147^ carbon then the sp^3^ carbon content will increase over time. Furthermore, when the ion energies are at the optimum, it will lead to the formation of the clusters inside the substrate which will further slow down the etching of the diamond nuclei. Finally, the effect of hydrogen radicals are also important for etching the non-diamond carbon. The etching rate by hydrogen radicals is an order of magnitude higher for non-diamond carbon than sp^3^ bonded carbon. The combined effect of etching by hydrogen radicals and the optimum energy of the impinging ions leads to formation of stable diamond nuclei (bottom panel Fig. 11). The whole process has been demonstrated by schematic in Fig. 11. Similar models were also proposed by Gerber *et al.*^142^ and Kulisch *et al.*^143^

**Fig. 12:**
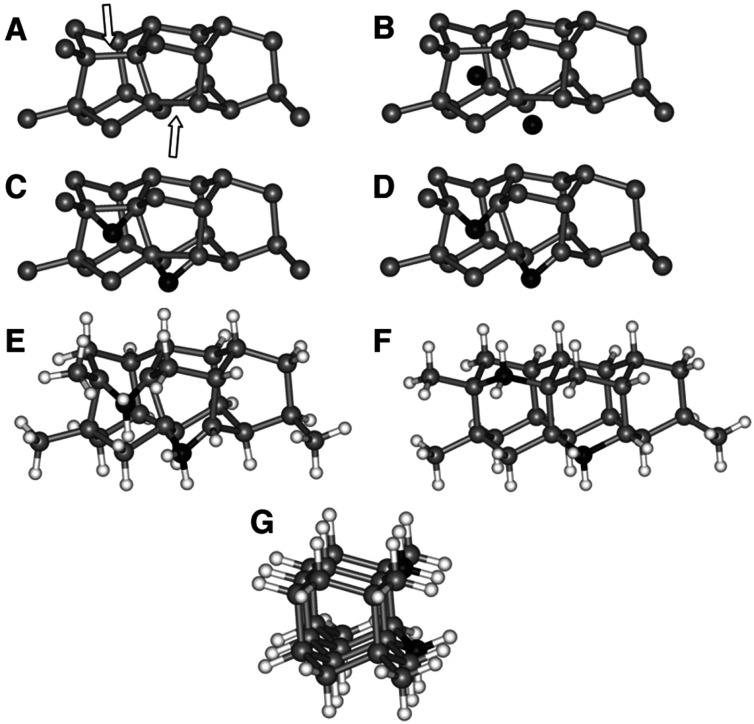
The figure shows the pathway of conversion of diamond-like clusters to diamond clusters. The structures have been derived from simulations. (Reprinted with permission from Lifshitz *et al.*, *Science*, 2002, **297**, 1531, AAAS.^144^)

**Fig. 13:**
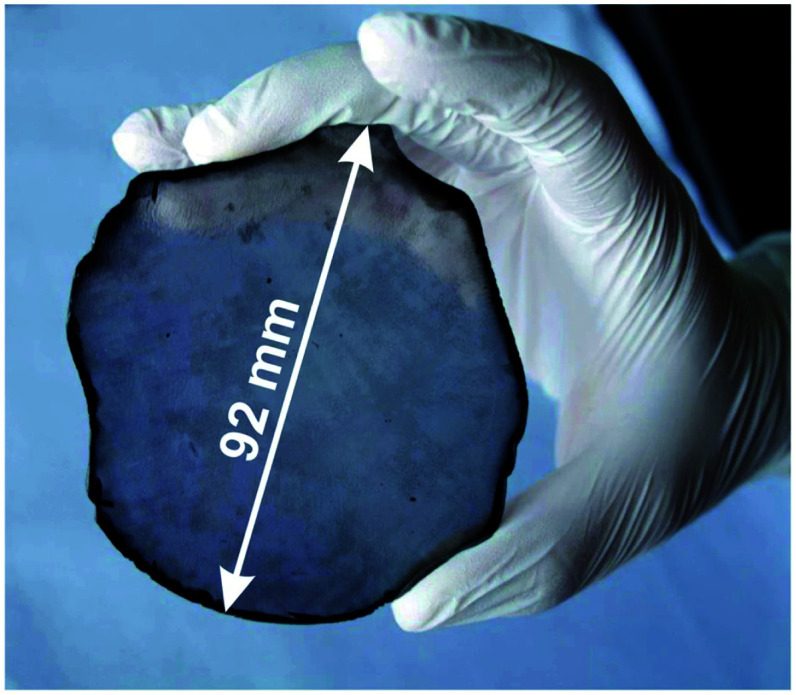
A large single crystal diamond wafer grown on Ir substrate using BEN as nucleation technique. (Reprinted from Schreck *et al.*, *Sci. Rep.*, 2017, **7**, 44462, under a Creative Commons Attribution 4.0 International License.^36^)

Lifshitz *et al.*^144^ proposed an alternative model for BEN. They said nucleation is a bulk process rather than a surface process. Also, they assumed that diamond nuclei can form under conditions where it is thermodynamically unfavourable in comparison to non-diamond carbon. According to them the nucleation process advances through the following steps. (I) A dense amorphous hydrogenated carbon (a-C:H) layer of ∼1 to 2 nm thickness is formed. The formation of this layer is facilitated by subplantation of energetic carbon, hydrocarbon and hydrogen species on the substrate surface. (II) Pure sp^3^ carbon clusters, containing tens of atoms, precipitates spontaneously in the a-C:H phase. The precipitation is induced by the thermal spikes caused by the energetic species impinging on the substrate surface. Only a small fraction (∼1 in 10^5^) of all clusters are diamonds and the rest are amorphous carbon. Simulations by the authors have shown that a tetrahedral arrangement of C atoms exists in these clusters with occasional small diamond crystallites embedded inside. The formation of the sp^3^ clusters is dependent on the concentration of hydrogen in the clusters. Hydrogen free a:C and low concentration a-C:H clusters do not yield any sp^3^ crystallites according to the simulations. (III) The faults in defective clusters are annealed through incorporation of interstitial carbon and hydrogen termination. The pathway for the conversion of diamond-like clusters to diamond clusters is shown in Fig. 12. The defective clusters have reactive sites as indicated by arrows in the figure where C atoms are added. These C atoms attach to neighbouring atoms while old bonds are broken and eliminated. The dangling bonds in the cluster are terminated by adding hydrogen atoms forming a diamond cluster. (IV) The diamond clusters grow several nanometers in size by the conversion of amorphous carbon to diamond at the amorphous carbon–diamond interface. This process is facilitated by the energetic hydrogen ions displacing loosely bound amorphous carbon to a new diamond position. Lifshitz *et al.*^144^ did their simulations for nucleation of diamond on silicons substrates. More recently, Schreck *et al.*^36^ extended this theory for iridium substrates. They were successful in growing a large single crystal wafer (∼90 mm diameter) weighing 155 carats using BEN as nucleation technique. Fig. 13 shows the large single crystal plate grown by Schreck *et al.*^36^

**Fig. 14:**
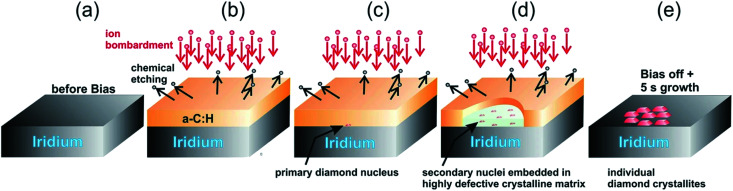
Schematic of the IBI-BLG model. Note the lateral expansion of the thinner domain along with the formation of secondary nuclei in panel d. (Reprinted from Schreck *et al.*, *Sci. Rep.*, 2017, **7**, 44462, under a Creative Commons Attribution 4.0 International License.^36^)

The model given by Lifshitz *et al.*^144^ can explain nucleation using BEN for nanocrystalline diamond on silicon substrates. This model is also true for primary nucleation generation on Ir substrates as well but according to Schreck *et al.*^36^ this is not enough to explain large lateral domain formation over few microns within minutes, that too without any twinning or secondary nucleation. They suggested a new model called Ion Bombardment Induced-Buried Lateral Growth (IBI-BLG). The schematic of the model suggested is shown in Fig. 14a–e. The initial three steps (Fig. 14a–c) are similar to what was suggested by Lifshitz *et al.*^144^ The fourth step in IBI-BLG explains the lateral growth of defective diamond crystal domains. These defective crystal domains are thinner than the surrounding aC:H layer as shown in Fig. 14d. Even in the defective crystal domains, only the carbon atoms close to Ir surface acts as nucleation site. The rest of the layer is etched away once the bias is removed and diamond growth condition is achieved (Fig. 14e). The lateral growth of the domain happens at the boundary between the defective crystal domain and the aC:H layer. The growth is mainly driven by ion bombardment at the interface between these domains. In earlier work Zaiser *et al.*^148^ showed that ionic bombardment can be used to transform non-diamond carbon into crystalline diamond under appropriate conditions. Using a similar argument Schreck *et al.*^36^ then explained the conversion of aC:H to diamond like structures at the interface between the domains. They concluded that for every carbon atom that is embedded at the interface by ballistic transport the domain grows by two carbon cells. The new carbon at the interface increases the local carbon density as well as provides the kinetic energy for the relaxation of a few carbon atoms at the interface into the diamond domain thus increasing its size. Once the bias voltage is removed all the carbon not close to the Ir surface are etched away and heteroepitaxial diamond growth starts.

BEN so far is the only technique that can induce nuclei and nucleation sites *in situ* in the growth chamber. The typical nucleation density achieved by BEN is of the order of 10^10^ cm^−2^ but Yugo *et al.*^149^ claimed that a nucleation density of 10^14^ cm^−2^ is possible and the suppression in density is primarily caused by insufficient delivery of source gas. But, such a high density is not possible with the critical nucleus size estimated by Yugo *et al.*^149^ They estimated that a critical nucleus will have 10^3^ carbon atoms. This is equivalent to a carbon sphere of 2.2 nm in diameter. This gives a possible density of ∼10^13^cm^−2^ (see Fig. 1). Nonetheless, before the demonstration of high seeding density by diamond nanoparticle, BEN was an effective technique to achieve high nucleation density. It has been used to grow diamond on variety of materials,^150–156^ selective area growth^157–159^ and heteroepitaxial growth.^36,160–163^

### 2.3 Chemical nucleation

**Fig. 15:**
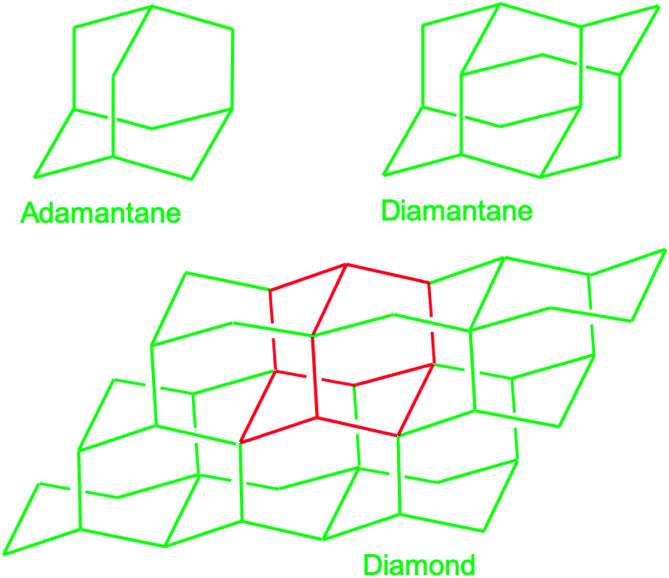
The diamond cage along with first two members of the diamondoid family are shown here. The red lines marked in the diamond cage shows how the smallest member of the diamondoid family sits on the cage.

In this subsection chemical nucleation of diamond films will be discussed. We will discuss seeding by a class of molecules called diamondoids. They are mostly found in petroleum products and the largest ever synthesised diamondoid is anti-tetramantane.^164^ Larger diamondoids which have also been called Nanometer-Sized Diamond Molecules have been isolated from petroleum products.^165,166^Fig. 15 shows the cage structure of the diamond lattice along with the structure of the first two members of the diamondoid family. The red lines in the diamond cage shows how the adamantane molecule sits in the diamond cage. Matsumoto and Matsui^167^ in 1983 and then Olah^168^ in 1990 suggested the use of caged molecules as possible seeds for diamond growth. The main motivation being that with the small size of diamondoid molecule (1 nm) it would be possible to grow ultrathin layer of diamond. As discussed in the Introduction (Fig. 1), it is possible to get very high seeding density with seeds of the size of 1 nm, though it would not be possible to see the seeds with conventional techniques like AFM. Techniques like spectral ellipsometry^53^ may be able to reveal the presence of monolayer of diamondoid on substrates. Another application that was thought of was to create diamond with custom colour centres. The idea being that a cage like structure would be chemically synthesised containing a nitrogen, silicon or other elements at the centre of the cage. These elements would then act as vacancy centre once the diamondoid seed is used to grow nanometer size diamond crystal.

**Fig. 16:**
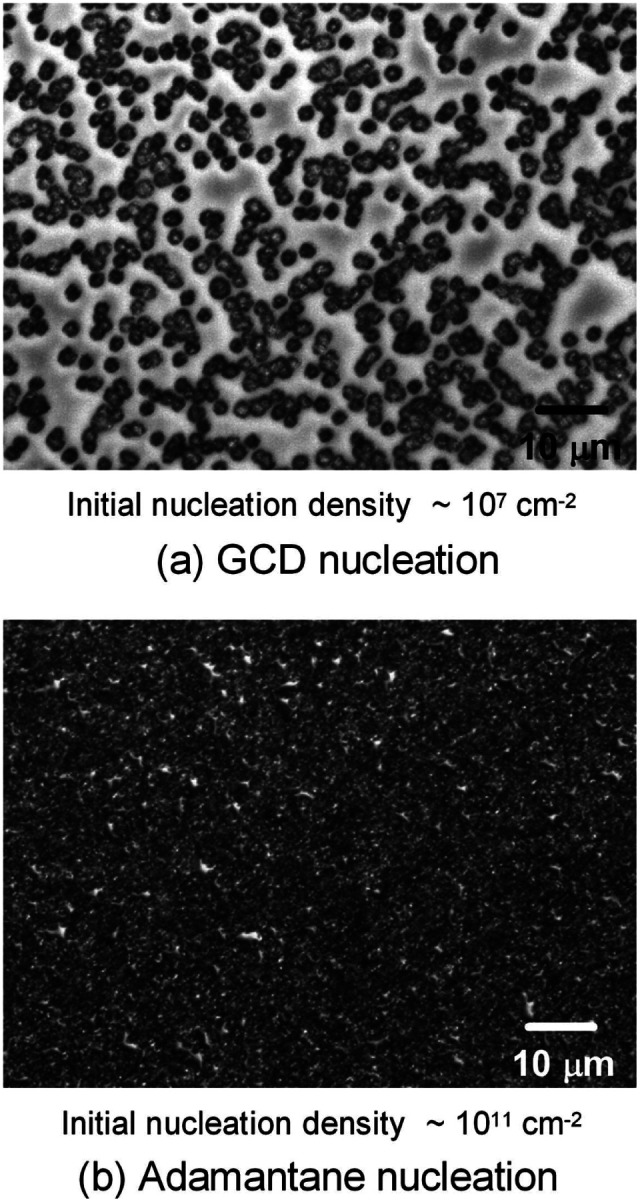
Images of diamond films grown after seeding with (a) graphite cluster diamond (b) adamantane seeding. (Reprinted with permission from Tsugawa *et al.*, *J. Phys. Chem. C*, 2010, **114**, 3822, American Chemical Society.^169^)

**Fig. 17:**
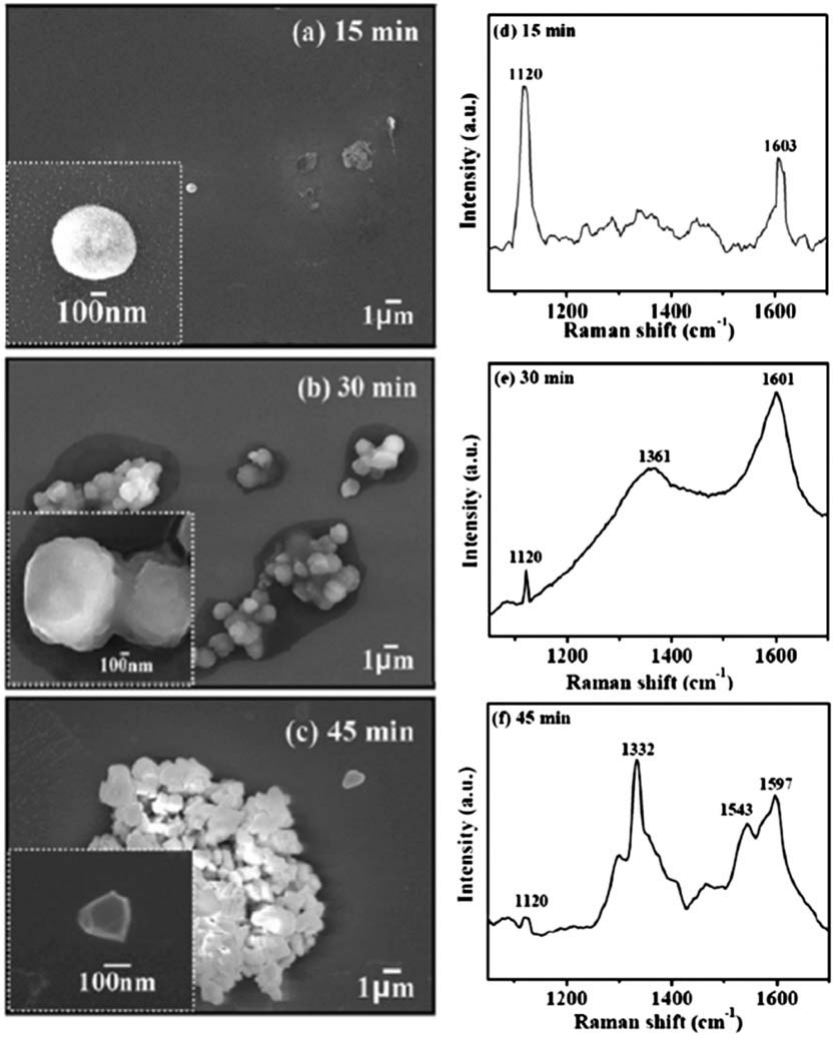
SEM images and Raman spectroscopy after growth on adamantane coated Si wafer for varying durations. Panel A shows the formation of tiny particles after 15 min of growth. The corresponding Raman spectra in Panel D shows no signs of diamond. The inset shows one of the particle like features. The first appearance of diamond peak in Raman spectra is seen after 45 min of growth. (Reprinted with permission from Tiwari *et al.*, *J. Appl. Phys.*, 2010, **107**, 103305, AIP Publishing.^170^)

**Fig. 18:**
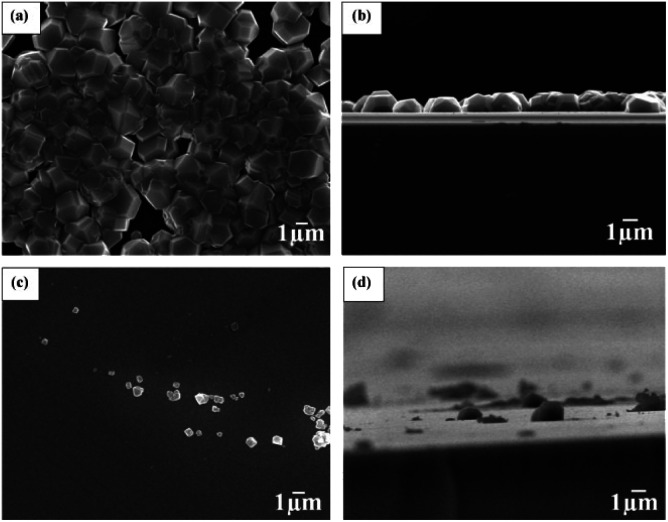
SEM images of diamond grown on adamantane coated and non-coated substrates. A clear enhancement in seed density is seen in Panel A when compared with panel C. Panel A is adamantane coated substrate. Panel B and D are the cross-sectional view of the films shown in Panels A and C respectively. (Reprinted with permission from Tiwari *et al.*, *Chem. Eng. J.*, 2010, **158**, 641, Elsevier.^171^)

In 2010 Tsugawa *et al.*^169^ studied the enhanced nucleation of diamond by adamantane on Si wafers. The adamantane powder was dissolved in hexane or ethanol and the substrate was sonicated in the solution prior to deposition. After sonication the substrates were washed in ethanol. For comparison with adamantane seeding, separate pieces of the substrates were seeded with graphite cluster diamond (GCD) particles. The films were grown using CVD process at 150 °C. The optical images of the films are shown in Fig. 16a (GCD) and b (Adamantane). It is evident from the images that the use of adamantane solution enhanced the seeding density by orders of magnitude. In the same year Tiwari *et al.*^170–172^ attempted to grow diamond on silicon coated adamantane which was directly sublimated on the substrate. They deposited a 0.9 mm thick layer of diamondoid prior to growth. The film was grown using CVD at around 530 °C. The methane concentration during these growths were kept at 0.6%. In general it is accepted that high methane concentration in the initial stages of growth helps to quickly grow and protect the seeds in the harsh plasma environment. The SEM images along with micro Raman spectroscopy of the sample after 15, 30 and 45 min of growth are shown in Fig. 17a, b and c respectively. One can see some seed formation on the surface of the substrate but a dense coverage is missing. In the Raman spectroscopy the characteristic diamond peak at 1332 cm^−1^ is only seen after 45 min of growth. The absence of large scale seeding can be attributed to high temperature of growth, which is higher than the sublimation temperature of adamantane, and also the low methane concentration in the gas mixture. The SEM images of diamonds grown with and without adamantane coating are shown in Fig. 18a–d. Fig. 18a and b show the image of the diamond film grown on adamantane coated substrate. The images for non-coated samples are shown in Fig. 18c and d. A clear enhancement in seeding density was evident from the thick diamond films grown on the seeded substrate when compared with unseeded substrates. Even then a uniform coverage was not achieved as was evident from the pinholes present in thick diamond films as shown in Fig. 18a.

**Fig. 19:**
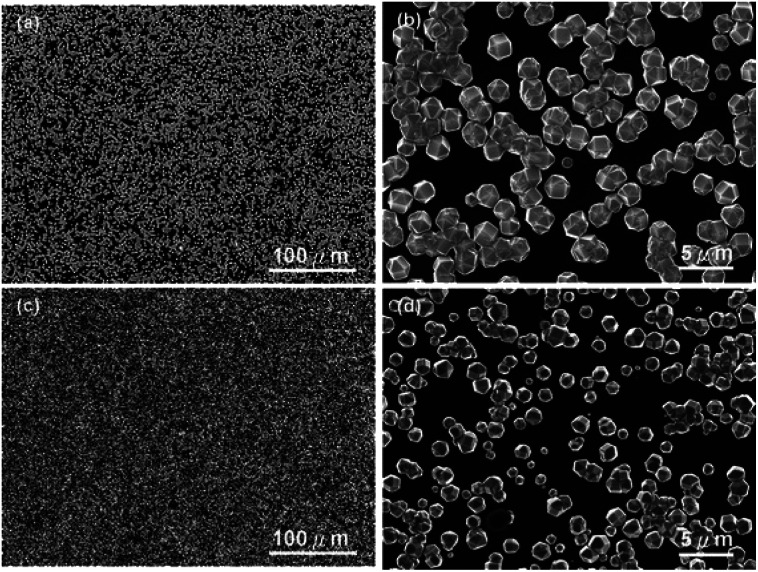
SEM of diamond on Si seeded with adamantane solution. The top two panels are for growth with adamantane in ethylene glycol and the bottom two are for adamantane in diethylene glycol. (Reprinted with permission from Chen *et al.*, *RSC Adv.*, 2013, **3**, 1514, Royal Society of Chemistry.^174^)

**Fig. 20:**
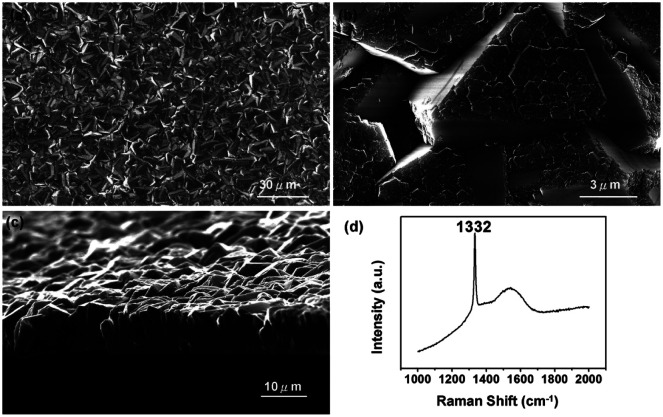
SEM of diamond on sapphire seeded with adamantane solution. The growth duration for this film was 48 h. Panel D shows the Raman spectra of the film with a clear hump at 1550 cm^−1^ indicating the presence of some graphitic carbon. (Reprinted with permission from Chen *et al.*, *RSC Adv.*, 2014, **4**, 18945, Royal Society of Chemistry.^175^)

In an alternative approach Tiwari *et al.*^173^ used a platinum surface coated with adamantane for diamond growth. In this case the adamantane was not sublimated to the Pt surface. Instead, the Pt coated substrates were immersed in an adamantane solution made by dissolving the diamondoid in hexane. After seeding the substrates were introduced in a CVD system with 1% methane in hydrogen. The growth temperature was kept close to 700 °C. From the Raman spectroscopy of samples that were grown for up to 15 min, no 1332 cm^−1^ characteristic diamond peak was seen. The calculated seeding density from the first few minute growth samples was around ∼10^7^ to 10^8^ cm^−2^. A similar approach was adapted by Chen *et al.* for growth of diamond on silicon^174^ and sapphire.^175^ They dissolved the adamantane in ethylene glycol and diethylene glycol. The seeded substrates were then introduced into a CVD system for diamond growth. The gas mixture for growth on silicon was 2% methane in hydrogen. The temperature of growth was around 600 °C. SEM images of diamond on silicon after one hour of growth are shown in Fig. 19a–d. Fig. 19a and b are for silicon seeded with adamantane dissolved in ethylene glycol and Fig. 19c and d are for adamantane dissolved in diethylene glycol respectively. In both cases no large scale films were obtained. The seed density as calculated from the SEM images were around ∼1.4 to 3.4 × 10^8^ cm^−2^. Similar results were obtained for diamond grown on sapphire. The authors had also reported a thick diamond film grown on sapphire^175^ (see Fig. 20a–c). A careful examination of the SEM image in Fig. 20c shows the presence of some void like patterns even after 48 h of growth. Furthermore the Raman data in Fig. 20d shows a clear hump around 1550 cm^−1^ indicating the presence of some graphitic carbon. Such graphitic carbon is generally not seen in films with high seeding density.^176^ If we compare the diamond growth by Chen *et al.*^174,175^ with that of Tiwari *et al.*^173^ discussed above, a marginal increase in seeding density can be seen. However, this can be attributed to the solvent^177^ even though in the case of Chen *et al.*^174,175^ they do not see in any noticeable enhancement in seeding due to the solvents. In both the cases we see seeding density enhancement over spontaneous seeding but the enhancements are not enough to get pinhole free thin diamond films.

**Fig. 21:**
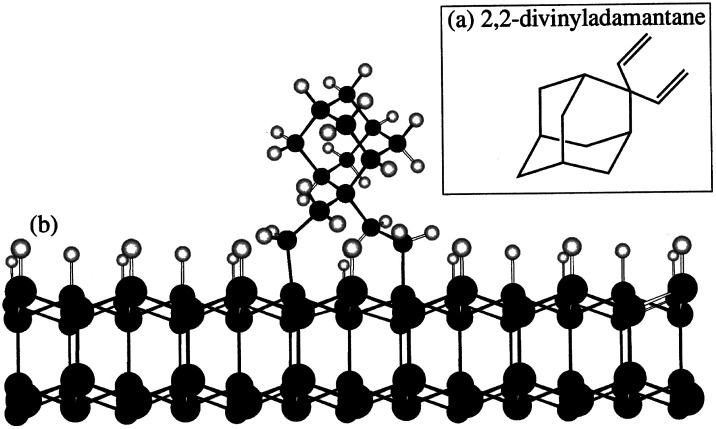
(a) 2,2-Divinyladamantane (b) schematic of DVA molecule attached to silicon surface. (Reprinted with permission from Leroy *et al.*, *Appl. Phys. Lett.*, 1998, **73**, 1050, AIP Publishing.^180^)

In all the studies^169–175^ discussed so far the smallest member of the diamondoid family was either directly sublimated or deposited on the substrates by dissolving in a solvent. Except for Tsugawa *et al.*^169^ the growth temperatures were also well above the sublimation temperature of adamantane (∼250 °C). There was no chemical bonding between the substrate and the molecule. As a result it is quite possible that the high temperature of the growth process sublimates the adamantane seeds leading to low seeding density. This can be overcome if it is possible to covalently attach the seed molecules to the silicon surface. In early 90s Linford *et al.*^178,179^ were able to show covalent attachment of organic molecules to silicon surface. A similar approach was used by Leroy *et al.*^180^ to attach 2,2-divinyladamantane (DVA) to silicon. The schematic of a DVA molecule attached to the silicon surface is shown in Fig. 21b. DVA is primarily an adamantane molecule with two alkene chains (Fig. 21a) which facilitates the attachment of the molecule to the silicon surface. Following this attachment technique Giraud *et al.*^181^ were able to show nucleation density of the order of 10^9^ cm^−2^. This is further improvement over what was seen for adamantane coated substrates, but it is still far from the seeding densities needed for pinhole free thin diamond films. The growth conditions used by Giraud *et al.*^181^ were 1% methane in hydrogen with 850 °C growth temperature. Even though in this case the molecule was covalently attached to the substrate, the seeding density is still low. This can be due to high temperature used during growth of diamond.

**Fig. 22:**
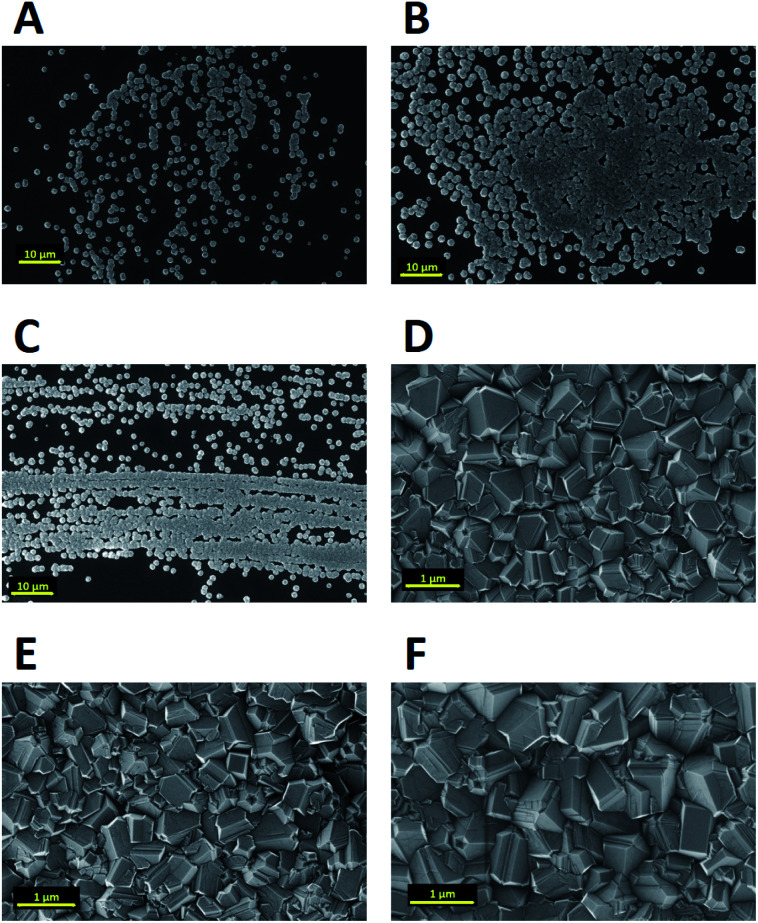
Diamond grown on silicon wafers seeded with DVA after 0–60 min of incubation. It was observed that after 30 min of incubation (Panel D) a pinhole free diamond layer could be achieved. (Reprinted with permission from Mandal *et al.*, *ACS Appl. Mater. Interfaces*, 2016, **8**, 26220, American Chemical Society.^176^)

Mandal *et al.*^176^ using the same strategy of covalently bonding DVA to silicon substrates, introduced a low temperature incubation step during diamond growth. The low temperature incubation step was done at 250 °C and since the molecule is covalently attached to the substrate it was expected that the seed molecule will survive this temperature. Fig. 22 shows the diamond films after varying incubation period. It was observed that after 30 min of incubation a pinhole free diamond layer could be achieved. The seed density calculated from AFM data in this work was around 6 × 10^11^ cm^−2^. The point to be noted here is that in this study, as well, large area films could not be fabricated. This is due to imperfect grafting of DVA on the silicon surface. The key step in this study which made the high density seeding possible, as opposed to the work by Giraud *et al.*,^181^ is the low temperature incubation step. For growth of thin diamond films using CVD, it is important to have a low temperature growth step while seeding with diamondoid molecules for CVD growth of diamond. This step helps the survival of the chemical precursor.

Low temperature growth was also attempted by Tsugawa *et al.*^169^ using adamantane seeds only. The diamond was analysed by Raman spectroscopy with 244 nm excitations (UV) and UV excitations are not suitable for collecting information about non-diamond carbon.^182^ Hence it is difficult to conclude anything on the quality of the diamond layer in the study by Tsugawa *et al.*^169^ Furthermore in studies where solvents have been used to dissolve the diamondoid molecules, it is important to see if the solvent themselves can lead to nucleation enhancements. Mandal *et al.*^176^ found that limited enhancement in nucleation density can be seen for certain solvents. Recently, higher diamondoids have been used to create vacancy centres in diamond.^183,184^ Zhang *et al.*^183^ used 7-dichlorophosphoryl[1(2,3)4]pentamantane dissolved in toluene to grow small diamond crystals. During the growth they introduced silane in very low quantities to create silicon vacancy centres. Furthermore, diamondoids have been used to grow nanodiamonds^185–188^ using HPHT technique. The discussion on such growth is beyond the scope of this review.

### 2.4 Nucleation through surface damage

**Fig. 23:**
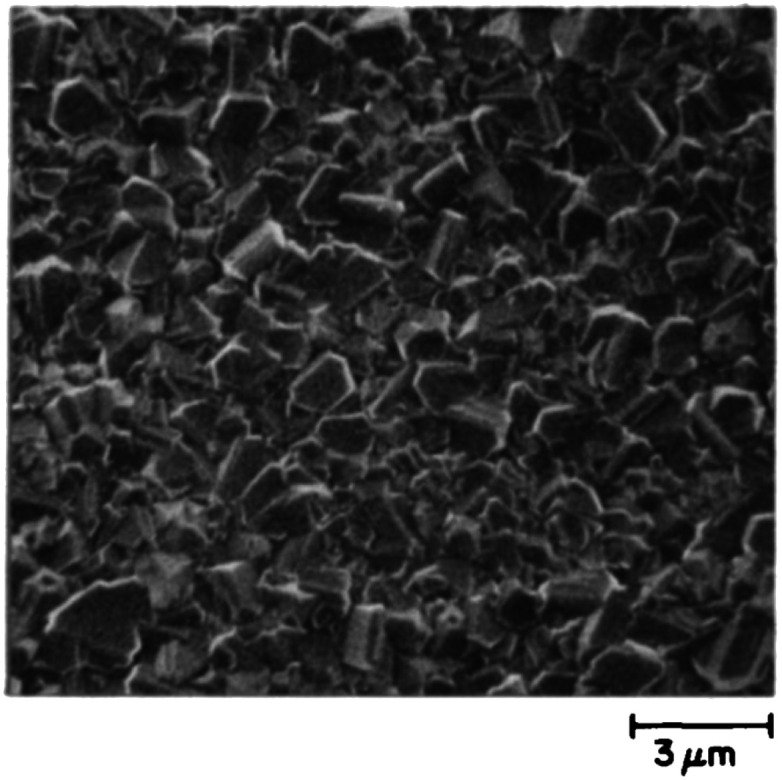
Boron doped diamond film grown on scratched silicon surface. (Reprinted with permission from Fujimori *et al.*, *Vacuum*, 1986, **36**, 99, Elsevier.^189^)

In this section two very closely related techniques will be discussed. One is known as mechanical scratching and the other is called ultrasonic particle treatment or micro chipping. The idea being to create micro/nano damage on the substrate surface. In 1981 Spitsyn *et al.*^33^ reported growth of diamond on foreign substrates. It was reported that spontaneous nucleation on foreign substrates were mostly seen on defects like scratches, grain boundaries, dislocations *etc.* Even though this work mentioned nucleation enhancement through scratches, it is not clear if the scratches were intentionally introduced to create nucleation sites. From the reading of the history of CVD in the early years written by John Angus,^34^ it is clear that lot of secrecy was maintained around diamond growth by both the American and Soviet researchers. The earliest record of mechanical scratching was probably reported by Fujimori *et al.*^189^ They were able to grow boron doped diamond on silicon wafers. The silicon wafers were polished with No. 1500 diamond powder. No. 1500 refers to 1500 mesh diamond powder which has a size range of 8–15 μm. The diamond film was continuous and a SEM image of the film is shown in Fig. 23.

**Fig. 24:**
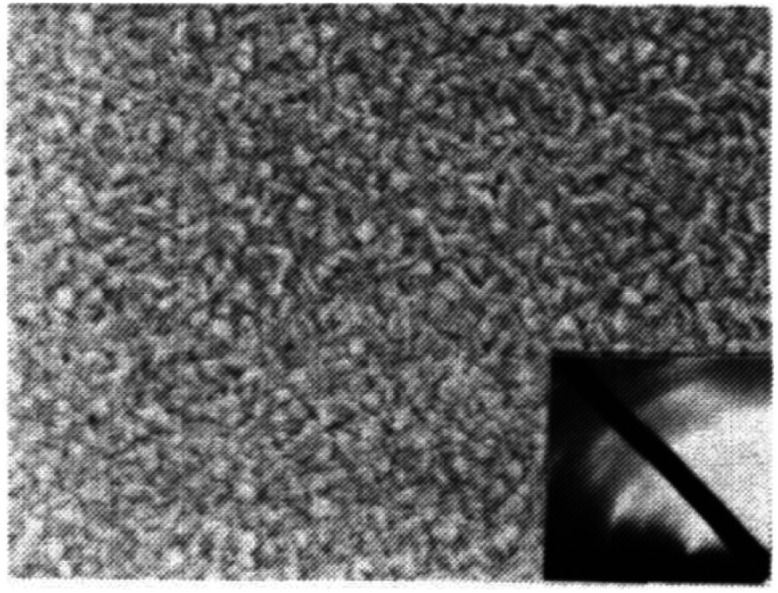
Diamond film grown by CVD on sonicated silicon substrate. The inset shows the RHEED pattern of the grown film. (Reprinted with permission from Yugo *et al.*, MRS Online Proceedings Library, 1987, **97**, 327, Springer Nature.^190^)

Yugo *et al.*^190^ experimented with various substrate treatments, primarily to roughen the surface. One of them was hand polishing of the substrate with diamond paste containing 5–10 μm diamond particles. The pressure applied during polishing was categorised as weak hand pressure and strong hand pressure. They found that weak hand pressure was better for nucleation concluding, that small defects on the substrate surface helped in enhancing nucleation of diamond. They also subjected the silicon surface to supersonic waves in alcohol/diamond slurry containing 5–10 μm particles. A thin film of diamond was grown on the sonicated silicon substrate by CVD. The SEM of the diamond film is shown in Fig. 24. The damage caused by the sonication process was considered to be less than 10 nm in size. This was due to non-visibility of any surface damage with SEM at 30k magnification.

Chang *et al.*^191^ studied various surfaces for growth of diamond. They roughened the surfaces to investigate enhancement in nucleation. A polished silicon surface was gently abraded with diamond powder containing particles less than 0.5 μm in size. Visible scratches could be observed under SEM but no diamond crystallites could be seen lodged in the substrate. No enhancement in nucleation was found by this treatment. A second scratching method was employed by Chang *et al.*^191^ In this case the surface was scratched by the same diamond powder sandwiched between two silicon substrates. Under SEM small diamond particles could be seen lodged on the silicon surface. A semi-continuous film was produced on silicon scratched by the sandwich method. One thing to note here is that there is no quantification of the applied force (weak hand pressure, strong hand pressure, gentle *etc.*) for abrading both by Yugo *et al.*^190^ and Chang *et al.*^191^ This experiment in a way confirmed that diamond growth due to abrasion is primarily from the lodged diamond particles on the substrate, but, contaminants can also enhance nucleation. For example, Chang *et al.*^191^ saw chains of diamond formation, preferentially on the contaminated sites, when scratched with stainless steel. Iijima *et al.*^192,193^ studied the early stages of diamond formation on substrates abraded with 10–40 μm diamond particles in water. It was found that small flakes of diamond, few tens of nanometers in size, were lodged on the silicon surface. It is interesting to note that the flakes were three orders of magnitude smaller than the abrading particles in water. After seeding, a short growth was done on the substrates. As expected, no continuous film was seen after the short growth but the diamond flakes survived the growth conditions and small diamond crystals were observed. The seed density was estimated to be in the range of 10^10^ to 10^11^ cm^−2^.

**Fig. 25:**
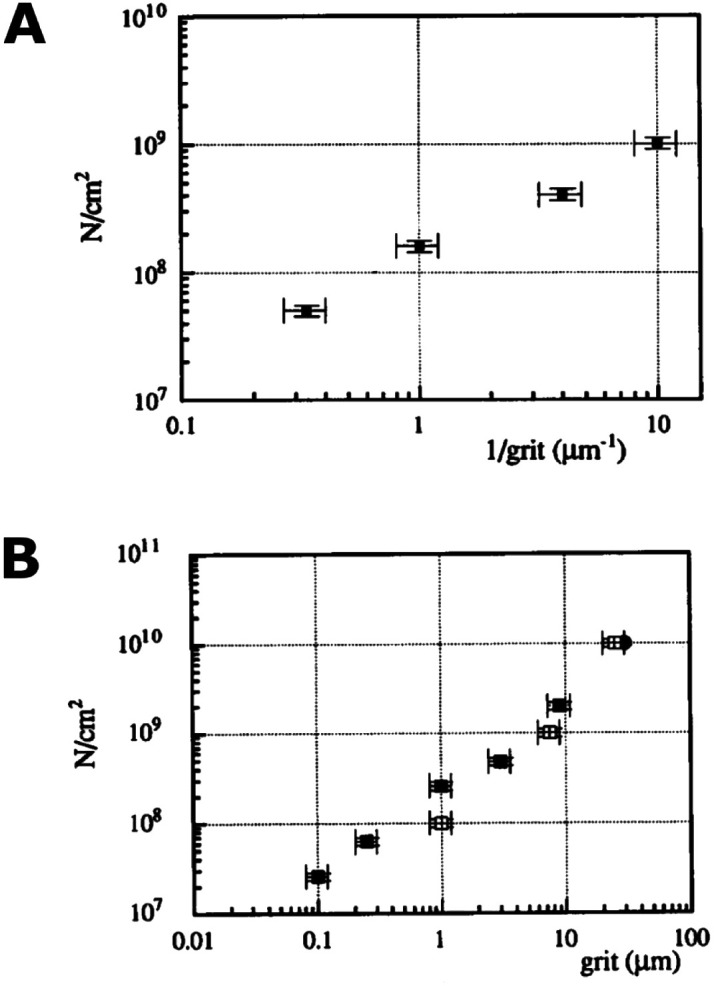
Panel A: Nucleation density as a function of inverse grit size for abrasive polished substrate. Panel B: Nucleation density as a function of mean particle size in diamond solution used for ultrasonic pretreatment. Note the difference in the *x*-axis of the two graphs. (Reprinted with permission from Ascarelli *et al.*, *Appl. Surf. Sci.*, 1993, **64**, 307, Elsevier.^194^)

Scratching experiments by polishing in diamond grit conducted by Yugo *et al.*^190^ and Chang *et al.*^191^ had different results. In one case there was enhancement in seeding^190^ and in another no such enhancement (polishing not sandwich method, see earlier) was seen.^191^ The difference in the two experiments was the grit size. Ascarelli *et al.*^194^ studied the effects of grit size on nucleation for scratching by polishing as well as ultrasonic treatment. The nucleation density variation with grit size is given in Fig. 25. Panel A in the figure shows nucleation density as a function of inverse of grit size for polished substrates. Panel B shows the nucleation density as a function of grit size for sonicated substrates. Note the difference in *x*-axis between the panels. It was found that the nucleation density being inversely proportional to the grit size for polished surfaces. In contrast, for ultrasonic seeding the density was directly proportional to the grit size. The explanation for this was postulated as follows. When the substrate surface is scratched, it produces broken bonds. The number of these broken bonds is proportional to the energy dissipated (*E*_d_) in the process. If the total load and time during scratching is kept constant then the energy dissipated will be constant irrespective of the grit size. The dissipation of this energy takes place in a narrow region close to the surface. The thickness of this region is proportional to the grit size *G*. If we assume *E*_c_ as the condensation energy per unit volume and S is the surface area of the substrate then 
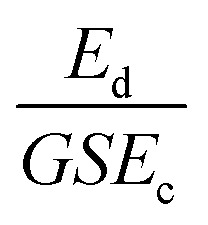
 is a number proportional to the nucleation density. Since *E*_d_, *S* and *E*_c_ stays same during the experiment, so nucleation density is proportional to 1/*G* and this is what is seen experimentally in Fig. 25A. For the ultrasonic case, similar bond breaking events happen due to collision of the particles with the substrate surface. The energy delivered to the substrate surface is proportional to the kinetic energy of the particle which will be delivered over a small area that will come in contact with the surface. For a particle of mass *M* and a contact area of *C*, nucleation density is proportional to 
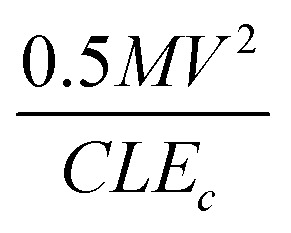
, where *V* is the velocity of the particle at the surface, *E*_c_ is the condensation energy per unit volume and *L* is the material dependent length scale over which the energy is dissipated. Here the assumption has been made that the contact area is proportional to *G*^2^ (*G*: grit size) and the mass would be proportional to *G*^3^. Assuming all other parameters staying constant the number of nucleation sites is directly proportional to *G* as is seen in Fig. 25B. The results are slightly different if both mechanical and ultrasonic scratching are used one after another. Smolin *et al.*^195^ saw an increase in nucleation density on molybdenum substrates with decrease in grit size. In this case prior to ultrasonic treatment, the molybdenum substrates were polished with 0.5 μm diamond grit. To increase nucleation density with small particles, Akhvlediani *et al.*^196^ had suggested a technique called “hammering” technique. In this technique small diamond particles (<0.25 μm) are mixed with larger particles (>2 μm, diamond and non-diamond) in the slurry. The bigger particles impart momentum to the smaller particles thus increasing the probability of damage and incorporation on the substrate by the smaller particles. By optimising the technique nucleation, densities in excess of 5 × 10^10^ cm^−2^ can be achieved.

**Fig. 26:**
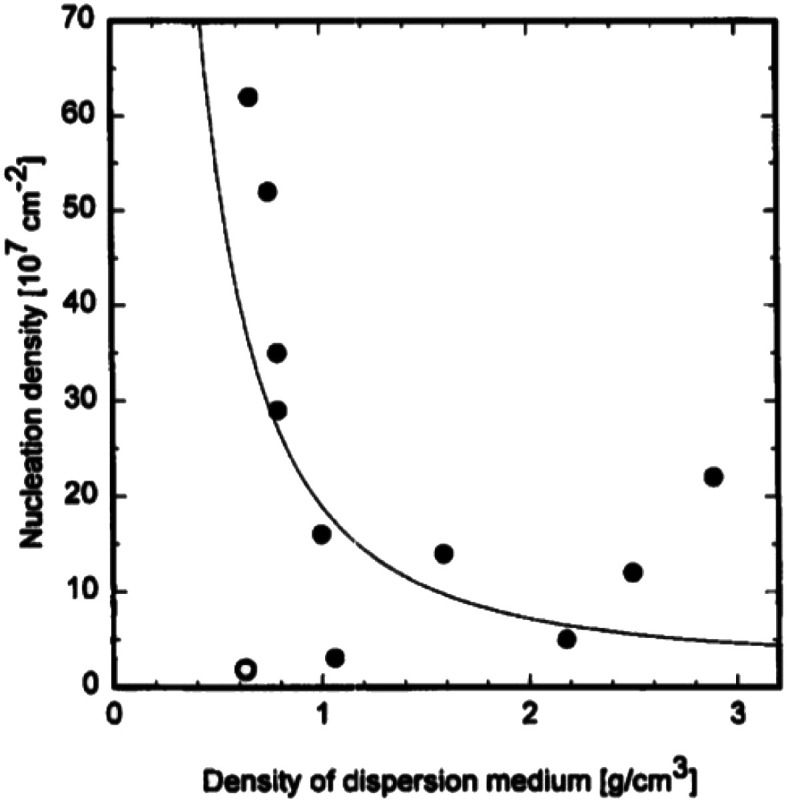
Variation of nucleation density with density of dispersant media. (Reprinted with permission from Schweitz *et al.*, *Diamond Relat. Mater.*, 1996, **5**, 206, Elsevier.^202^)

The explanation given above assumes any nucleation enhancement seen as solely due to scratching. It is to be noted that high nucleation density in earlier experiments by Chang *et al.*^191^ were only observed when small bits of diamond were lodged on the surface. Furthermore, researchers have also tried to abrade substrates with metallic and ceramic grits but had failed to see any nucleation enhancement.^197–199^ One exception to this is abrading silicon with silicon carbide where nucleation enhancement has been observed.^200,201^ In the results discussed so far the dispersion medium has been water or alcohol. The effect of dispersion medium on nucleation density was reported by Schweitz *et al.*^202^ They used 11 different media with densities ranging between 0.6 and 2.9 g cm^−3^. Ultrasonic waves passing through these liquids will create jet-streams which are responsible for accelerating suspended particles in the solution. A correlation between dispersant density and nucleation density was found. Fig. 26 shows the variation of nucleation density with dispersant media density. From ultrasonic theory it seems obvious that less dense media will impart more velocity in the diamond particles, this means higher number of particles reaching the substrate surface will have critical velocity to create bond breakage. Intuitively this should lead to higher nucleation density for low density media. This trend is seen in the data shown in Fig. 26 with the exception of pentane. We should also keep in mind that the dispersant media, used in these experiments, in itself can enhance nucleation density as has been discussed in the chemical nucleation section.

Okubo *et al.*^203^ and Ihara *et al.*^204^ studied the correlation between the residual diamond after scratching and the nucleation density. If we consider *N*_s_ as the residual diamond density and *N*_D_ as the nucleation density, it was found that *N*_s_ was several orders of magnitude higher than *N*_D_. This was attributed to the etching away of diamond residue in hydrogen plasma during the initial stages of growth. Researchers^205–208^ have successfully tackled this problem by depositing an interlayer on the scratched surface resulting in nucleation densities close to residual diamond density. The narrative in this section has continuously shifted between damage created by polishing and ultrasonic particle treatment, where the main idea is the enhancement of nucleation density by surface damage creation and residual diamond. The serious draw back of this technique is the damaged/rough substrate. This will introduce roughness at the interface between diamond and the substrate. The rough diamond film is non-ideal for applications like MEMS,^209,210^ thermal management^211^*etc.* While the ultrasonic scratching technique can be applied to 3D surfaces, the mechanical polishing is limited to only planar materials. This technique is now commonly used for growing diamond on ceramics^212–219^ for various applications though electrostatic seeding have also been used on ceramics.^105,106^

### 2.5 Interlayer driven nucleation

**Fig. 27:**
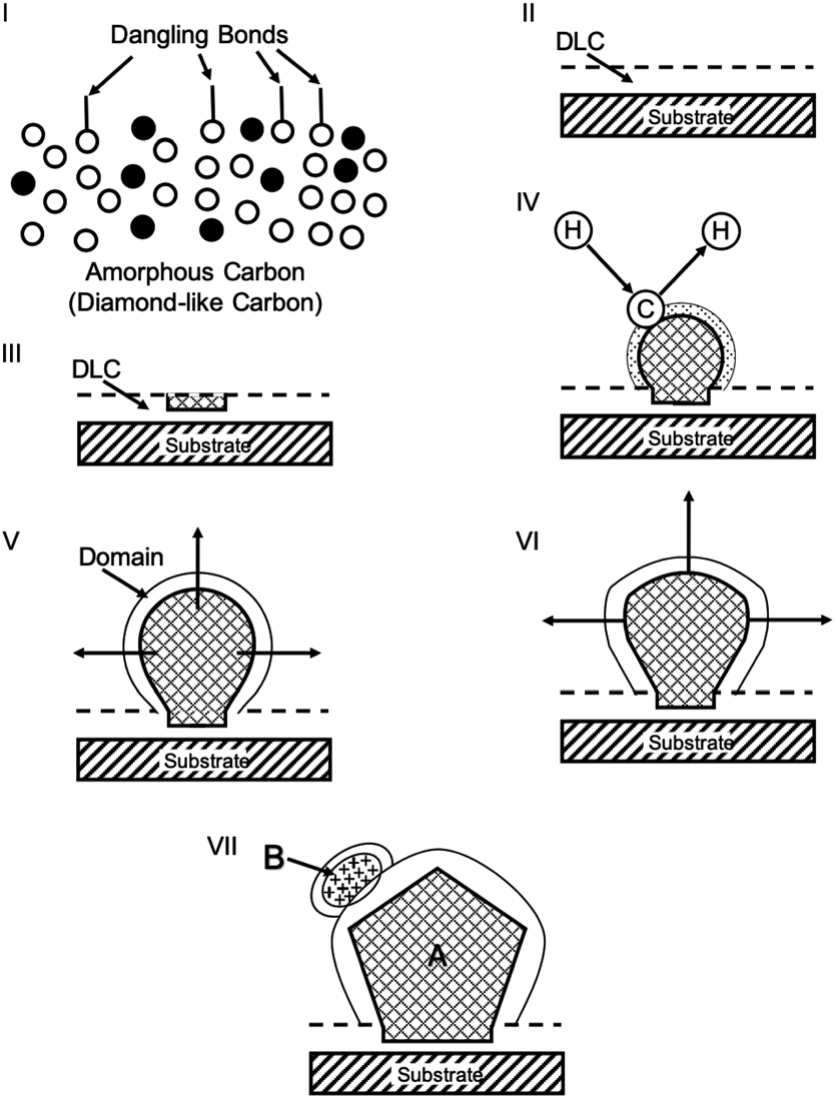
Schematic describing various steps of diamond growth assisted by DLC. (Reprinted with permission from Singh *J. Mater. Sci.*, 1994, **29**, 2761, Springer Nature.^221^)

In this section nucleation enhancement by non-diamond carbon, carbides or carbide forming interlayers will be discussed. All these interlayers have been shown to assist the nucleation of diamond. Ravi *et al.*^220^ reported the enhancement of nucleation density through pre-deposition of diamond like carbon (DLC) layer on scratched molybdenum surfaces. They attributed the enhancement in density to solid-state conversion of DLC to diamond. Later on Singh^221^ studied the mechanism of enhancement by growing diamond on copper grids. It was found that 2–5 nm diamond crystallites were embedded in a 8–14 nm of diamond-like amorphous carbon layer after growth. Fig. 27 shows the schematic of the steps in the diamond growth assisted by DLC layer. The growth mechanism follows the following steps. (i) Formation of carbon clusters on the substrate surface. (ii) Non-diamond carbon is converted to more stable sp^3^ bonded carbon network (DLC) assisted by faster etching of non-diamond carbon. (iii) Crossover from disorder sp^3^ network (DLC) to diamond. (iv–vi) Diamond growth takes place on the small crystals formed within DLC and finally, (vii) secondary nucleation occurs on the surface of enlarged diamond crystals. This model basically describes the conversion of DLC into diamond.

Similarly, graphite can also lead to enhancement in nucleation for diamond growth. Ong *et al.*^222^ saw this kind of enhancement when graphitic layers were formed on copper through carbon implantation. Morrish *et al.*^206^ tried a variety of treatment for nucleation enhancement on molybdenum surface. Enhancement was seen when the surface was coated with hydrocarbon oil or 10–20 nm of evaporated carbon. Nucleation enhancement has also been achieved by depositing a layer of C_60_ and C_70_ molecules,^223^ carbon fibre treated silicon surfaces,^224^ carbon nanotube on silicon,^225^ glassy carbon on silicon,^177^ amorphous carbon,^205,207^ fingerprints on substrate^206^*etc.* Polycarbynes, a carbon based network polymers,^226^ has also be used for nucleation enhancement with limited success.^227–229^ The seeding density has been limited to 10^7^ to 10^9^ cm^−2^.^230^ Sun *et al.*^231,232^ used poly(phenylcarbyne) which was converted to diamond using CVD process. Looking at the results in the work it can be said only individual crystals were formed. Chen *et al.*^230^ used poly(phenylcarbynes) to enhance diamond nucleation density on scratched silicon.

Kobayashi *et al.*^233^ showed nucleation enhancement on substrates coated with iron. The iron layer promoted the diffusion of carbon into the substrate thus promoting diamond nucleation. Lux *et al.*^234^ described nucleation on carbide forming metals on the basis of carbon diffusion rates in the metal carbides. The carbon from the plasma reacts with the metal and forms carbide. The carbide growth rate and the rate of diamond nucleation is dependent on carbon diffusion rate through the carbide. The lower the diffusion rate, the faster the nucleation rate. For example hafnium and tantalum have very low carbon diffusion rates and diamond nuclei are formed very quickly on these metals.^235^ In contrast niobium with intermediate diffusion constant has very distinct characteristic. Diamond nuclei formed on the surface can dissolve in the metal, under CVD conditions, leading to decrease in number of nuclei with time after the initial nuclei formation. More recently, Li *et al.*^236^ studied the effects of thin nickel, aluminium and nickel–aluminium layer on diamond nucleation. They deposited the layers on silicon and observed nucleation enhancement on nickel surface. The addition of aluminium to nickel suppressed the formation of graphitic carbon and increased the purity of diamond though aluminium layer in itself did not result in good quality diamond.

As stated earlier, diffusion of carbon through the carbides is dependent on thickness of carbide layer, substrate temperature, diffusion rate and availability of carbon at the carbon–carbide interface. As long as there is no nuclei the diffusion rate is dependent on the carbon reaching the surface from the plasma. The moment a nucleus is formed the concentration of carbon at the nucleus–substrate interface increases and so does the diffusion rate. If the rate of growth of this nucleus exceeds the diffusion rate at the carbon–carbide interface the nucleus will survive and grow to form large diamond crystal. For niobium, as mentioned before, under certain CVD conditions the growth rate of diamond can be reduced below the diffusion rate thus leading to dissolution of the formed nuclei. Similarly, other carbide forming metal layers like titanium, zirconium, vanadium, molybdenum, chromium, cobalt, nickel *etc.* can enhance nucleation. The key point being the formation of a carbon diffusion barrier so that the carbon from the plasma can stay close to the surface rather than get absorbed in the substrate layer. Direct deposition of carbides can also assist in diamond nucleation in a similar fashion as explained above. Even though enhancements are seen with carbon or carbon containing layers, it is not to the same level as electrostatic seeding, BEN or chemical nucleation.

### 2.6 Mixed technique

Mixed technique or combinatorial technique refers to the use of combination of above mentioned technique. One such technique was pioneered by Rotter *et al.*^67,68^ The full details of the technique were not revealed in the 1998 article,^67^ as a result not many researchers used the technique at the time. In this technique a thin layer of amorphous carbon is deposited on the substrate using normal diamond growth conditions in the CVD reactor. The carbon coated substrate is seeded in an ultrasonic bath by immersing in a diamond grit suspension. The second step is same as the ultrasonic seeding technique discussed in the nucleation through surface damage (micro chipping) section. The process of coating the substrate with carbon does not generate any diamond or nucleation sites. This step though can form carbide with the substrate where possible. This seeding technique was used by Philip *et al.*,^66^ as discussed above, to grow diamond films for mechanical and thermal property measurements. Sumant *et al.*^237^ used this seeding technique to fabricate very thin free standing diamond membranes but they modified the second step of the seeding technique. Instead of using micron sized grit for the ultrasonic step Sumant *et al.*^237^ used nanodiamond particle (4–10 nm) solution. The small size of the particle can be helpful in the formation of thin diamond films. More recently this process has been used by Mukherjee *et al.*^104^ for growing diamond on silicon carbide. Other mixed techniques have been the use of scratching in combination with carbon deposition or other metal deposition. For example Barnes *et al.*^207^ deposited amorphous carbon on scratched silicon surface. In doing so a seed density of 3 × 10^10^ cm^−2^ was achieved which is several orders higher than simply scratched silicon. Morrish *et al.*^206^ coated the scratched substrates with hydrocarbon oil and observed enhanced nucleation over scratched substrates.

## 3 Summary of different nucleation techniques

Diamond nucleation on foreign substrates by various techniques have been reviewed. At present the most widely used technique is electrostatic seeding for polycrystalline films, BEN for heteroepitaxial films and scratching for growth on ceramics even though electrostatic seeding has been used for ceramics. A brief summary of all the techniques are given below.

### 3.1 Electrostatic seeding

This is currently the most widely used seeding technique. This technique can be used for all forms of surfaces including 3D surfaces. A prior knowledge of the zeta potential of substrate helps in determining the type of seed solution (H-terminated or O-terminated) needed for high seed density on the substrate. A nucleation density in excess of 10^11^ cm^−2^ is needed for growth of thin (<50 nm) diamond films but thicker films can be grown with much reduced density. The nucleation density used for diamond growth should be tailored to the stability of final diamond/substrate structure. A high nucleation density is not a measure of the quality of the diamond films.

### 3.2 Bias enhanced nucleation

At present this is one of the few routes available for growing epitaxial films on non-diamond substrate. This is also the technique to create nucleation sites/nuclei *in situ* during the growth process. The main disadvantage of this technique is the requirement of a conductive substrate. Various models to explain nucleation by BEN has been discussed here. The latest model, IBI-BLG, explains the growth of large domains over several microns within minutes. This techniques has been used to grow large single crystal wafer (∼90 mm diameter) weighing 155 carats.

### 3.3 Chemical nucleation

Chemical nucleation mostly refers to nucleation driven by a class of molecules called diamondoids. For successful growth of fully coalesced diamond films it is essential to devise strategies to protect the small seeds during the initial stages of diamond growth. It has been shown that maintaining a low substrate temperature in the initial phases of the growth helps in the survival of the diamondoid molecules. It is also beneficial to covalently attach the diamondoid seeds to the substrate surface rather than spin coating the surface. While judging the nucleation enhancement due to diamondoid, the contribution from the solvents should also be considered.

### 3.4 Nucleation through surface damage

In this section two closely related techniques, mechanical scratching and micro chipping, have been discussed. The main aim is to create small damages on the substrate surface which then acts as nucleation sites. Mechanical scratching can be used only for planar surfaces as it involves polishing the substrate with diamond grit. On the other hand micro chipping, which is done in an ultrasonic bath by dipping the substrate in a diamond colloid, can be used for all forms of surfaces. It was found that the nucleation enhancement was driven by small diamond particles (tens of nanometers in size) lodged on the surface of the substrate. A major disadvantage of the technique is the interface roughness between diamond film and substrate making the grown diamond film unsuitable for MEMS or thermal management purposes. This technique is commonly used for growing diamond on ceramics.

### 3.5 Interlayer driven nucleation

Non-diamond carbon, carbides or carbide forming layers can also enhance diamond nucleation. These can include any non-diamond carbon containing material that will assist the nucleation of diamond. Researchers have seen enhancements from fullerenes, carbon fibre, carbon nanotubes, glassy carbon, polycarbynes, hydrocarbon oil, finger prints to name a few. However, this does not include polymers loaded with nanodiamond. It was found that anything that can create a carbon diffusion barrier on the substrate surface can assist in nucleation enhancement.

### 3.6 Mixed technique

Mixed techniques refer to process where a combination of process are used to enhance nucleation sites. One such approach is to deposit a small amount of carbon on the substrate before exposing it to a diamond solution. Researchers have also seen nucleation enhancement when a scratched substrate has been coated with carbon or oil *etc.*

### 3.7 Comparison between commonly used techniques

Here a comparison is given between most commonly used techniques for diamond seeding/nucleation. Electrostatic seeding, BEN and seeding by surface damage are compared in Table 1. Chemical nucleation and interlayer diffusion techniques are not frequently used and hence not included in the table. The main reason for these techniques being not so popular is its inability to create high seed/nucleation density. If we compare the rest of the four techniques side by side, electrostatic seeding is the simplest one. The main disadvantage of this technique is the colloid itself which is delicate in nature. The preparation of the colloid for seeding is also non-trivial. However, the availability of commercial seed solutions, with well defined particle size and *ζ*-potential, reduces the challenges in this technique. For growth of epitaxial films on non-diamond substrates BEN is one of the available routes. This is the only technique that creates nuclei *in situ*. But this technique has major disadvantages, that it can be used for 2D conductive surfaces only. That rules out growth on all ceramic surfaces. For such surfaces seeding by surface damage or electrostatic seeing can be used. Surface damage can be created by polishing with diamond particle or by micro-chipping in ultrasound. The polishing can only be used on 2D surfaces but the microchipping can also be used on 3D surfaces. If the seed/nuclei densities are compared, BEN and surface damage gives densities close to 10^10^/cm^−2^. In comparison, electrostatic seeding can give seed densities in excess of 10^11^/cm^−2^. Finally, for many applications the interface between diamond and substrate is important. Roughness at the interface may lead to inferior performances. While electrostatic seeding and BEN gives smooth interfaces, seeding by surface damage creates wafer damage and leaves large residual particles resulting in rough interface.

**Table 1:** Table comparing the most commonly used technique

Electrostatic	BEN	Surface damage	
		Mechanical scratching	Micro-chipping in ultrasound
• Nanodiamond particle in colloid	• *In situ* generation of nucleation sites by applied bias	• Mechanical abrasion with micron or sub-micron diamond powder	• Micron sized diamond slurry work by microchipping on substrate
• 2D and 3D surfaces	• 2D surfaces only	• 2D surfaces only	• 2D and 3D surfaces
• Seed density > 10^11^ cm^−2^	• Nuclei density >10^10^ cm^−2^	• Seed density ∼10^10^ cm^−2^	• Seed density ∼10^10^ cm^−2^
• Colloids are delicate	• Conductive substrates only	• Wafer damage, residual particle	• Wafer damage, residual particle

### 3.8 Outlook and perspectives

The study of nucleation of diamond on non-diamond substrates have been going on for last four decades. Starting with isolated diamond nanoparticles on silicon in the early days, it is possible to get coalesced diamond layers with thickness close to 10 nm. Electrostatic seeding, done by dipping the substrate in a diamond colloid, is the most commonly used technique for growth of this diamond films on variety of substrates. For growth of epitaxial diamond layers BEN is one of the preferred techniques and has been used for growing large single crystal wafer. These two techniques can be used for growing diamond on majority of substrates. However, study of seeding on newer surfaces is still needed. For example, diamond is an excellent candidate for thermal management in high power devices if thick layers can be directly grown on power device materials. Like before, diamond seed layer can be easily attached to theses surfaces, but making a thick layer stick requires some ingenuity at the seeding level itself. Another area of interest is the preparation of robust, stable and environment friendly colloid for seeding. The electrostatic seeding works due to the surface charges on the diamond nanoparticles. The reason for particular kind of charge (positive/negative) is still controversial and is being actively looked at by various groups.

Similarly, the production of large (2 inch diameter or more) defect free single crystal is still a challenge. So far the largest high purity diamond plates are either grown on diamond by CVD or by high pressure high temperature techniques. Such single crystal plates are expensive and the high cost makes them unattractive for most applications. This is where techniques like BEN are useful. More work is needed before large single crystals can be cheaply produced, thus making them cost effective for variety of applications. Finally, the area of seeding technique that is least developed and yet has the potential for considerable impact is chemical nucleation. Diamondoids are extremely promising molecules for diamond seeding. There are only few examples of growth in literature using diamondoids. They have the potential to be cleanroom compatible diamond seed solution. Last but not least, if a chemical technique is found to form higher diamondoids (>1 nm in size) from lower diamondoids it has the potential to revolutionise diamond seeding/nucleation.

## Conflicts of interest

There are no conflicts to declare.

## Supplementary Material
